# Identification of bacteria and fungi responsible for litter decomposition in desert steppes via combined DNA stable isotope probing

**DOI:** 10.3389/fmicb.2024.1353629

**Published:** 2024-03-08

**Authors:** He Ye, Nare Tu, Zhendan Wu, Shilong He, Yu Zhao, Mei Yue, Mei Hong

**Affiliations:** ^1^Key Laboratory of Soil Quality and Nutrient Resources, College of Grassland, Resources and Environment, Inner Mongolia Agricultural University, Hohhot, China; ^2^Key Laboratory of Agricultural Ecological Security and Green Development, Universities of Inner Mongolia Autonomous Region, Hohhot, China

**Keywords:** DNA-stable isotope probing, soil microorganisms, functional genes, litter decomposition, nitrogen deposition

## Abstract

**Introduction:**

Soil microorganisms play crucial roles in determining the fate of litter in desert steppes because their activities constitute a major component of the global carbon (C) cycle. Human activities lead to increased ecosystem nitrogen (N) deposition, which has unpredictable impacts on soil microorganism diversity and functions. Nowadays, it is necessary to further study the succession of these microorganisms in the process of litter decomposition in desert steppe, and explore the effect of N deposition on this process. This issue is particularly important to resolve because it contributes to the broader understanding of nutrient cycling processes in desert steppes.

**Methods:**

In this study, DNA stable isotope probing (DNA-SIP) was used to study changes in soil bacterial and fungal community composition and function during 8 weeks of culture of ^13^C-labeled litter in desert steppes.

**Results:**

The results were as follows: (1) *Actinomycetota*, *Pseudomonadota*, and *Ascomycota* are the main microorganisms involved in litter decomposition in desert steppes; (2) N deposition (50 kg ha^−1^ year^−1^) significantly increased the relative abundance of some microorganisms involved in the decomposition process; and (3) N deposition likely promotes litter decomposition in desert steppes by increasing the abundances of N cycles bacteria (usually carrying GH family functional genes).

**Discussion:**

These findings contribute to a deeper understanding of the C assimilation mechanisms associated with litter residue production, emphasizing the importance of extensive C utilization.

## 1 Introduction

The carbon (C) content in soil, which is threefold higher than the atmospheric content, plays a crucial role in the C cycle of terrestrial ecosystems and simultaneously supports ecosystem services (Frouz, 2018). Litter decomposition is the basic process of regulating nutrient cycling, especially C cycling in terrestrial ecosystems, and constitutes the first stage of soil humus formation (Błońska et al., 2021). Litter decomposition is controlled by its chemical composition, non-biological conditions, and biological activities (Pioli et al., 2020). Biological factors play a dominant role in the process of litter decomposition, and non-biological factors can have various effects on biological factors (Dong et al., 2021). Because soil microorganisms are the primary participants in litter decomposition, their abundance and functions determine decomposition rates (Barel et al., 2019).

Fungi are capable of producing various extracellular enzymes that facilitate the degradation of recalcitrant lignocelluloses; thus, they comprise the most active decomposers of complex plant biopolymers (Wang W. et al., 2020). Fungi are primarily involved in the utilization of lignocelluloses and decompose such compounds into smaller molecules, which can then be used by bacteria (Miao et al., 2022). The numerous bacterial strains isolated from soil are capable of cellulose degradation. There is evidence that the abundance of soil bacteria gradually increases during litter decomposition, suggesting that the role of bacteria in litter decomposition processes has been underestimated (Dong et al., 2021). Thus, there is a need to explore the role of soil microbial communities in the biological mechanisms underlying litter decomposition.

Desert steppes, climatogenic ecotones located in arid ecosystems, constitute typical steppes. These are important grasslands in Eurasia; they comprising ~11% of Inner Mongolia’s steppe area (Qiu et al., 2022). Their characteristics include low vegetation coverage, low rainfall, and poor soil quality (Wang et al., 2022). They are currently in a period of severe degradation due to climate change and increased anthropogenic activities such as extensive agriculture and industry (Ma et al., 2022). Litter decomposition is an important factor of nutrient return in desert steppes; its decomposition process controls nutrients and the C cycle (Hoorens et al., 2003). In recent years, human activities have led to a tendency for increasing atmospheric nitrogen (N) deposition in China. From 2011 to 2015, the average estimated N deposition in this area was ~20 kg ha^−1^ year^−1^ (Liu et al., 2013; Yu et al., 2019). Continued atmospheric deposition of N has intensified with anthropogenic activities and has also been observed in dry lands (Ma et al., 2022). Although there is a lack of direct evidence to explain the mechanisms by which N addition influences litter decomposition, several hypotheses have been proposed: enzyme inhibition, copiotrophic, N-mining, and ecological stoichiometry (Finn et al., 2015). Additionally, a 4 years study showed that N deposition significantly promoted litter decomposition in desert steppes (Ye et al., 2022). In N-limited regions, the addition of small or moderate amounts of N can accelerate litter decomposition. However, N addition above a certain threshold can inhibit litter decomposition (Yin et al., 2022).

N deposition can lead to changes in nutrient uptake and photosynthetic efficiency by plants, ultimately affecting the C:N ratio of plant litter; importantly, litter with higher N content and lower C:N ratio decomposes faster (Song et al., 2017). Low quality leaf litter with high C:N ratio and low base cation content support the fungal-dominated energy channel with slow nutrient turnover while high-quality litter with low C:N ratio and high base cation content support the bacterial-controlled channel with fast decomposition and nutrient release (Wardle et al., 2004; Pietsch et al., 2014). Soil bacteria are mostly associated with decomposition of easily degradable compounds such as starch or glucose (Fierer et al., 2007), while soil fungi are important for decomposition of more recalcitrant compounds such as lignin (Urbanová et al., 2015; Algora Gallardo et al., 2021). However, some studies have indicated that the effects of N addition on litter decomposition are driven by changes in soil microbial community composition, rather than changes in litter quality (Maaroufi et al., 2017). Atmospheric N deposition reportedly has negative effects on the growth, composition, and function of soil microorganisms in all terrestrial ecosystems; these effects increase according to the N deposition rate and duration (Zhang et al., 2018). However, desert steppes have experienced an extended duration of N limitation, and many uncertainties remain concerning the effects of N addition on soil microorganisms in desert steppes (Wang H. et al., 2020; Jia et al., 2021). The relative abundances of specific soil microbial phyla are influenced by N addition, favoring the preferential metabolic activities of copiotrophic microorganisms with respect to labile organic C, rather than the participation of oligotrophic microorganisms in recalcitrant organic C metabolism (Fierer et al., 2012; Finn et al., 2015). Thus far, it is unclear which soil microorganisms are involved in litter decomposition in desert steppes; the effects of N deposition on these microorganisms are also unknown.

Because more than 99% of microorganisms cannot be cultured, the ability to directly link microbial phylogeny with functionality is limited (Miao et al., 2022). The DNA stable isotope probing (DNA-SIP), a new method that does not rely on traditional culture approaches, can be used to elucidate relationships between microbial activity (function) and characteristics in environmental samples (Ziels et al., 2018). DNA-SIP relies on the incorporation of heavy isotopes in microbial DNA during growth on labeled substrates, thus acting as a ‘filter’ to enrich the DNA of active populations (Chen and Murrell, 2010). To investigate the bacterial and fungal communities involved in litter decomposition in desert steppes and their response to N deposition, we applied ^13^C-litter to soil, then traced ^13^C in bacterial and fungal communities at the genus level using ^13^C-DNA-SIP. The specific objectives were (1) to identify the active bacterial and fungal taxa involved in litter decomposition in desert steppes and their succession at various stages of decomposition, (2) to evaluate how N deposition influences litter-utilizing bacterial and fungal communities, (3) to determine how participation in functional changes within bacterial and fungal communities is related to litter decomposition via metagenomic analysis, and (4) to explore microbiological mechanisms underlying the effects of N deposition on litter decomposition in desert steppes.

## 2 Materials and methods

### 2.1 Experimental site

The experiment was performed at Siziwang Banner (41°46′43.6″ N, 111°53′41.7″ E; elevation: 1456 m), in Inner Mongolia, Northern China. The average annual precipitation in the region is approximately 280 mm, and precipitation during the growing season (May to September) constitutes >70% of the total precipitation. The annual average temperature in the study region is 3.4°C. The soil in this region is classified as Haplic Calcisol (Food and Agricultural Organization Soil Classification System of the United Nations) and has a sandy loam texture. Soil pH (8.38), organic C (18.84 g kg^−1^), total N (1.88 g kg^−1^), NH_4_^+^-N (1.38 mg kg^−1^), NO_3_^−^-N (9.46 mg kg^−1^) and available P and K (5.88 and 281.31 mg kg^−1^, respectively) were initially determined. The plant community at the study site is dominated by *Stipa breviflora Griseb*.

### 2.2 Experimental design

**Figure 1:**
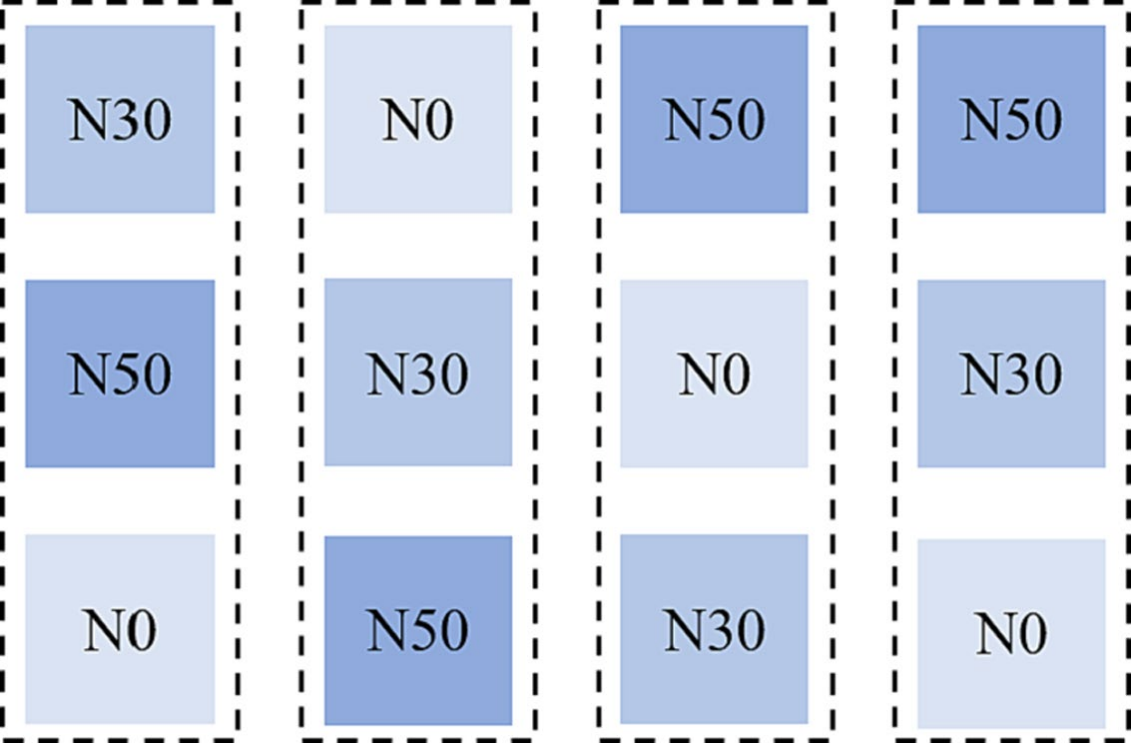
Experimental design. N0, control treatment; N30, N addition treatment (30 kg ha^−1^ year^−1^); N50, N addition treatment (50 kg ha^−1^ year^−1^).

Simulated N deposition experiments were established in December 2015. The amount of N added in this experiment was designed according to the amount of N deposition in this area and the amount of N added in similar experiments in the world. The N deposition in this area is ~15.00–17.00 kg N ha^−1^ year^−1^ (Zhang, 2021; Li, 2022). The experimental design included three N deposition conditions: control (N0, no N added), N addition of 30 kg ha^−1^ year^−1^ (N30), and N addition of 50 kg ha^−1^ year^−1^ (N50). The N deposition was simulated via wet deposition of NH_4_NO_3_ in the concentrated rainfall season from May to September. Specifically, NH_4_NO_3_ was mixed with purified muddy water and equally dispersed on each plot; the N0 treatment only received an equal amount of purified water. From October of the same year to April of the following year, NH_4_NO_3_ was mixed with the soil and evenly broadcasted by hand to simulate dry deposition; the same amount of soil was added to the N0 treatment. The monthly N application rate was determined by the percentage of the average monthly precipitation over the previous 5 years relative to the total annual precipitation (0.51, 1.17, 1.13, 2.82, 6.36, 18.25, 27.32, 13.04, 15.33, 6.91, 5.79, and 1.37% from January to December, respectively). These experiments were conducted using a randomized complete block design (Figure 1), with four replicates for each treatment. Each plot had an area of 7 m × 7 m, with 1 m spacing between plots.

### 2.3 ^13^C marker

In the peak season of vegetative growth during August 2021, an open space with flat terrain and uniform vegetation was selected in the experimental area. Six sample areas of 0.5 m (length) × 0.5 m (width) were established; three were used for ^13^C-pulse isotope labeling (labeled area) and three were used to calculate ^13^C natural abundance (unlabeled area). The minimum distance between sample areas was 2 m to limit interference by other factors related to pulse labeling. The dimensions of each labeling chamber were 0.5 m × 0.5 m × 0.5 m (L × W × H). The chamber was placed on the soil surface and sealed. Each chamber was equipped with an infrared gas analyzer to monitor the CO_2_ concentration. An air pump and an absorption bottle with NaOH were installed above the chamber to absorb CO_2_ in the atmosphere, and two mini fans were installed in the chamber. For each injection of ^13^CO_2_ (^13^C atomic abundance 99%), an electric fan was used to achieve thorough mixing, such that the CO_2_ concentration in the chamber remained in the range of 400–420 ppm. The labeling time was 8:00–17:00. After labeling had been completed, the labeling chamber was removed.

*S. breviflora*, a representative plant in desert steppes that constitutes ~65.11% of the total biomass, was collected from labeled and unlabeled areas. The aboveground part of the plant (beginning at 1 cm above the soil surface) was harvested, placed in a mesh bag, and transported to the laboratory. The collected samples were divided into two parts. One part was lyophilized and ground into powder; the δ^13^C values of ^13^C labeled and unlabeled plant samples were determined using an element analyzer connected to an isotope ratio mass spectrometer. The other part of the labeled plant material was used as a ^13^C-labeled substrate (i.e., ^13^C-labeled litter) in the culture experiments. The δ^13^C value of ^13^C-labeled litter was 431.09‰; the δ^13^C value of unlabeled litter was −24.90‰.

### 2.4 ^13^C-labeled litter decomposition test

The 0–10 cm soil samples were collected from 12 plots of N deposition, corresponding to four replicates for each of the three treatment types (N0, N30, and N50). For each treatment, soil samples were collected using the “five-point method,” and the four replicate samples for each N treatment type were thoroughly mixed. The soil samples were packed in glass bottles, each containing a dry weight of 400.00 g of soil. ^13^C-labeled litter (1.00 g) was added to each bottle; 1.00 g of unlabeled litter was added to the control. Deionized water was then added to the glass bottle until the soil moisture reached 40% of the field capacity. Destructive samples were collected at 0 (0 W), 1 (1 W), 2 (2 W), 4 (4 W), and 8 (8 W) weeks of culture.

### 2.5 DNA preparation and SIP gradient fractionation

Total DNA was extracted from each 0.5 g soil sample using a FastDNA spin kit for soil (MP Biomedicals, Cleveland, OH, United States), and DNA concentrations were determined using a NanoDrop ND-2000 ultraviolet-visible light spectrophotometer (NanoDrop Technologies, Wilmington, DE, United States). Genomic DNA (3 μg) was added to cesium chloride (CsCl) solution (buoyant density, 1.71 g mL^−1^) in an ultracentrifuge tube. An OptimaXPN-100 ultracentrifuge was then used to centrifuge each mixture at 45,000 rpm for 48 h. After centrifugation, the DNA was divided into 16 layers (approximately 290 μL per layer). The buoyant density of CsCl was measured for each layer. The fractionated DNA was precipitated with polyethylene glycol 6,000, washed twice with 70% ethanol, and re-dissolved in 30 μL of sterilized ultrapure water (Wang J. et al., 2020).

### 2.6 Real-time quantitative polymerase chain reaction

Bacterial 16S rRNA and fungal internal transcribed spacer (ITS) rRNA were analyzed via quantitative polymerase chain reaction (qPCR) on a Light-Cycler Roche 480 (Roche Molecular Systems, Pleasanton, CA, United States). Each 20 μL reaction contained 10 μL of 2× GoTaq^®^ qPCR Master Mix (Promega Biotech Co., Ltd., Beijing, China), 2 μL of DNA sample, 0.4 μL of each primer (10 mM), and 7.2 μL of DNA-free water. Purified amplicons were pooled in equimolar amounts and subjected to paired-end sequencing on an Illumina MiSeq PE300/NovaSeq PE250 platform (Illumina, San Diego, CA, United States), in accordance with standard protocols established by Majorbio Bio-Pharm Technology Co., Ltd. (Shanghai, China). Sequence data related to this study have been deposited in the National Center for Biotechnology Information (NCBI) database (Accession number: PRJNA1015017).

### 2.7 Shotgun metagenomic sequencing and analysis

Total genomic DNA (i.e., unstratified genomic DNA) was extracted from 0 W (initial time) soil samples using the E.Z.N.A.^®^ Soil DNA Kit (Omega Bio-tek, Norcross, GA, United States). This analysis was conducted via paired-end sequencing on an Illumina MiSeq PE300/NovaSeq PE250 platform (Illumina), in accordance with the standard protocols established by Majorbio Bio-Pharm Technology Co., Ltd. (Shanghai, China). Sequence data related to this study have been deposited in the NCBI database (Accession number: PRJNA979949).

## 3 Results

### 3.1 Bacterial and fungal DNA stratification analysis

**Figure 2:**
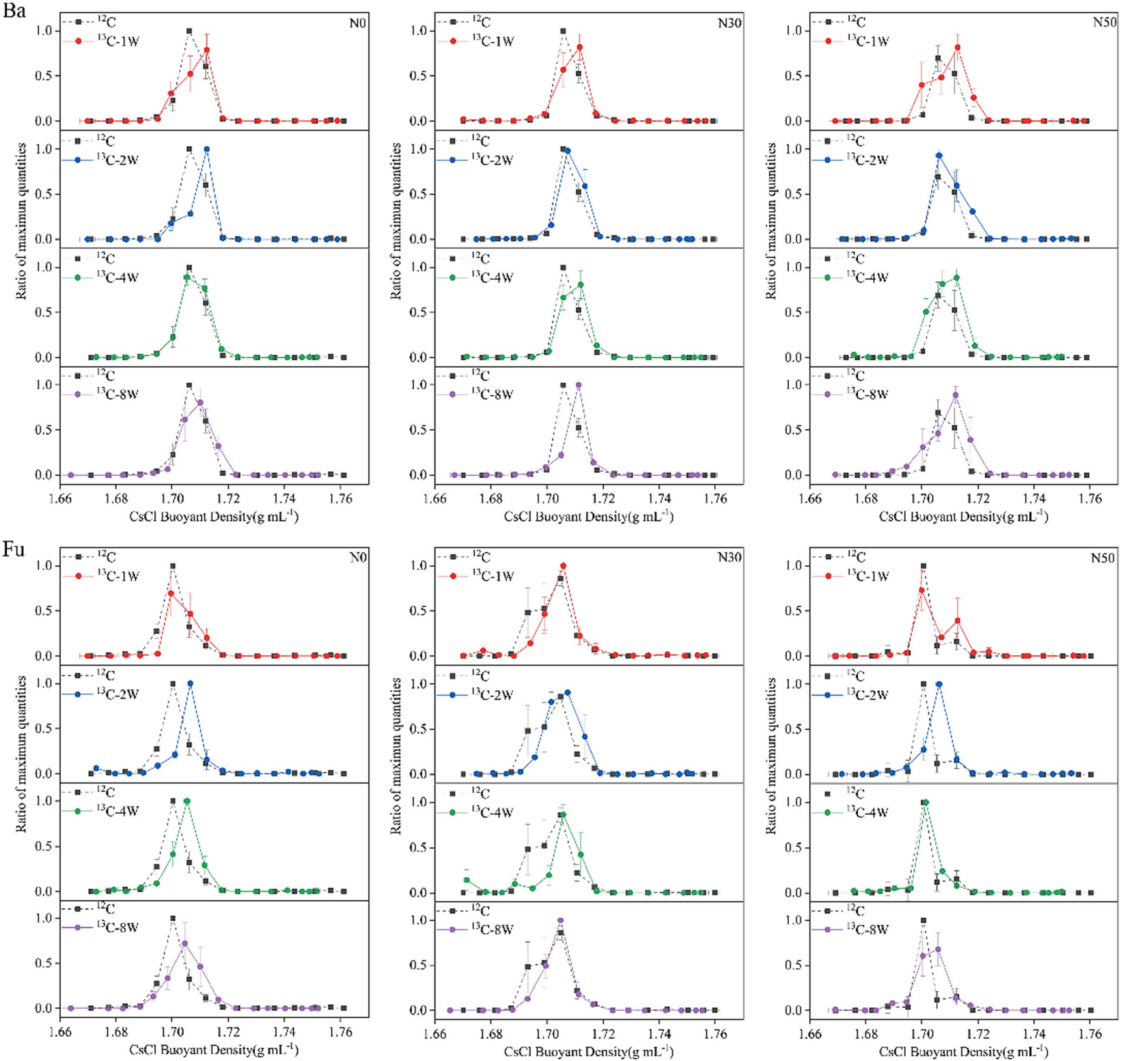
Relative abundances of bacterial (Ba) and fungal (Fu) DNA based on CsCl buoyant density values. N0, control treatment; N30, N addition treatment (30 kg ha^−1^ year^−1^); N50, N addition treatment (50 kg ha^−1^ year^−1^); ^12^C, control (litter decomposition at 0 weeks); ^13^C, ^13^C-litter; 1 W, 2 W, 4 W, and 8 W, litter decomposition at 1, 2, 4, and 8 weeks, respectively. Normalized data show the ratio of the gene copy number in each DNA gradient fraction to the highest abundance from each treatment. Whiskers denote the standard error of the mean (*n* = 3).

Sixteen gradient fractions of DNA (buoyant density, 1.6643–1.7638 g mL^−1^) from labeled (^13^C-litter) and unlabeled (^12^C-litter) samples were assayed by qPCR to determine the abundances of bacterial 16S and fungal ITS genes. In each N treatment, bacterial DNA from ^12^C-litter was enriched in the light fraction with a buoyant density of 1.7058–1.7061 g mL^−1^, whereas bacterial DNA from ^13^C-litter was enriched in the heavy fraction with a buoyant density of 1.7100–1.7189 g mL^−1^ (Figure 2, Ba). Bacterial DNA in heavy fractions (fractions, 8–9) from the ^12^C and ^13^C-litter microcosms was used for high-throughput sequencing. Fungal DNA from ^12^C-litter was enriched in the light fraction with a buoyant density of 1.7003–1.7048 g mL^−1^, whereas fungal DNA from ^13^C-litter was enriched in the heavy fraction with a buoyant density of 1.6995–1.7073 g mL^−1^ (Figure 2, Fu). Fungal DNA in heavy fractions (fractions 8–10) from the ^12^C and ^13^C-litter microcosms was used for high-throughput sequencing. Pairwise comparisons of bacterial and fungal samples in heavy fractions from labeled and unlabeled samples revealed significant differences along the first two principal coordinates [principal coordinates analysis (PCoA); Supplementary Figure S1], confirming successful labeling of the target microorganisms during incubation with ^13^C-litter. Therefore, we regarded microorganisms distributed in heavy fractions from ^13^C-litter microcosms as ^13^C-assimilating taxa.

### 3.2 ^13^C-assimilating bacterial and fungal community structure and diversity

**Figure 3:**
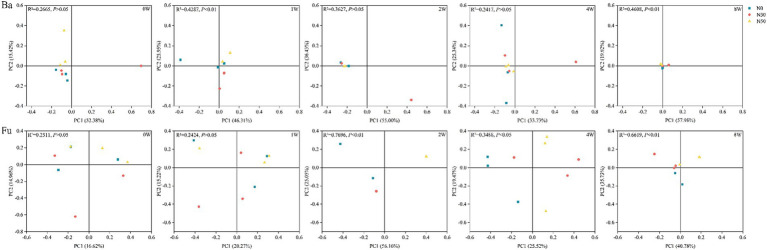
Principal coordinates analysis of ^13^C-labeled bacterial communities (Ba) and fungal communities (Fu) involved in litter decomposition based on operational taxonomic unit levels determined by abundance-based Jaccard distance. N0, control treatment; N30, N addition treatment (30 kg ha^−1^ year^−1^); N50, N addition treatment (50 kg ha^−1^ year^−1^); ^12^C, control (litter decomposition at 0 weeks); ^13^C, ^13^C-litter; 1 W, 2 W, 4 W, and 8 W, litter decomposition at 1, 2, 4, and 8 weeks, respectively.

Long-term N addition influenced ^13^C-labeled bacterial and fungal community structures and led to their convergence (PCoA; Figure 3). PERMANOVA analysis showed that the bacterial community structure significantly differed between N treatments at 1 W and 8 W (*p* < 0.05); the fungal community structure significantly differed among N treatments at 2 W, 4 W, and 8 W (*p* < 0.05). During litter decomposition, there were no significant differences in bacterial and fungal community diversity among N treatments (*p* > 0.05; Supplementary Figure S2). Decomposition increased bacterial and fungal community richness at 8 W (Chao1, Sobs), compared with the levels at 0 W (0 W, *p* < 0.05).

**Figure 4:**
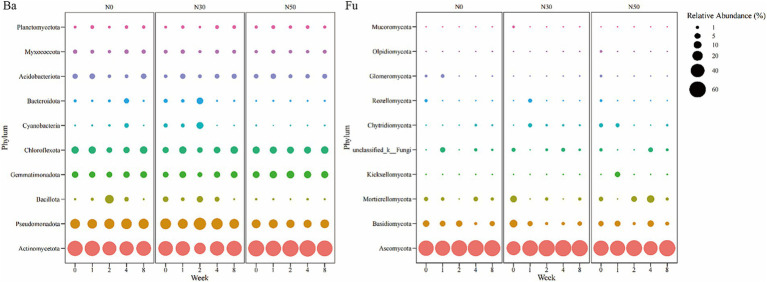
Bubble chart of relative abundances of ^13^C-assimilating bacteria and fungi (Phylum). Bacterial (Ba) and fungal (Fu) community composition represented by the most abundant phylum (top 10) in the heavy DNA fractions from the ^13^C-litter decomposition experiment. C, carbon; N, nitrogen; N0, control treatment; N30, N addition treatment (30 kg ha^−1^ year^−1^); N50, N addition treatment (50 kg ha^−1^ year^−1^); ^12^C, control (litter decomposition at 0 weeks); ^13^C, ^13^C-litter; 1 W, 2 W, 4 W, and 8 W, litter decomposition at 1, 2, 4, and 8 weeks, respectively.

Bacteria in the *Actinomycetota* (28.85–62.99%) dominated the heavy DNA fractions from 1 W–8 W of ^13^C-litter decomposition (Figure 4 Ba), followed by *Pseudomonadota* (12.56–32.87%), *Chloroflexota* (5.11–10.37%), *Gemmatimonadota* (3.45–10.81%), and *Acidobacteriota* (1.27–5.06%). The relative abundances of *Actinomycetota* in N0 and N30 treatments decreased from 0 W to 2 W and showed an increasing trend from 2 W to 8 W. In the N50 treatment, the lowest relative abundance of *Actinomycetota* appeared at 1 W. Fungi in the *Ascomycota* (91.76–94.64%) dominated the heavy DNA fractions at 8 W of ^13^C-litter decomposition (Figure 4 Fu). Notably, *Ascomycota* (67.27–83.18%) was the most abundant phylum at 0 W, implying that most *Ascomycota* fungi are not involved in litter decomposition.

**Figure 5:**
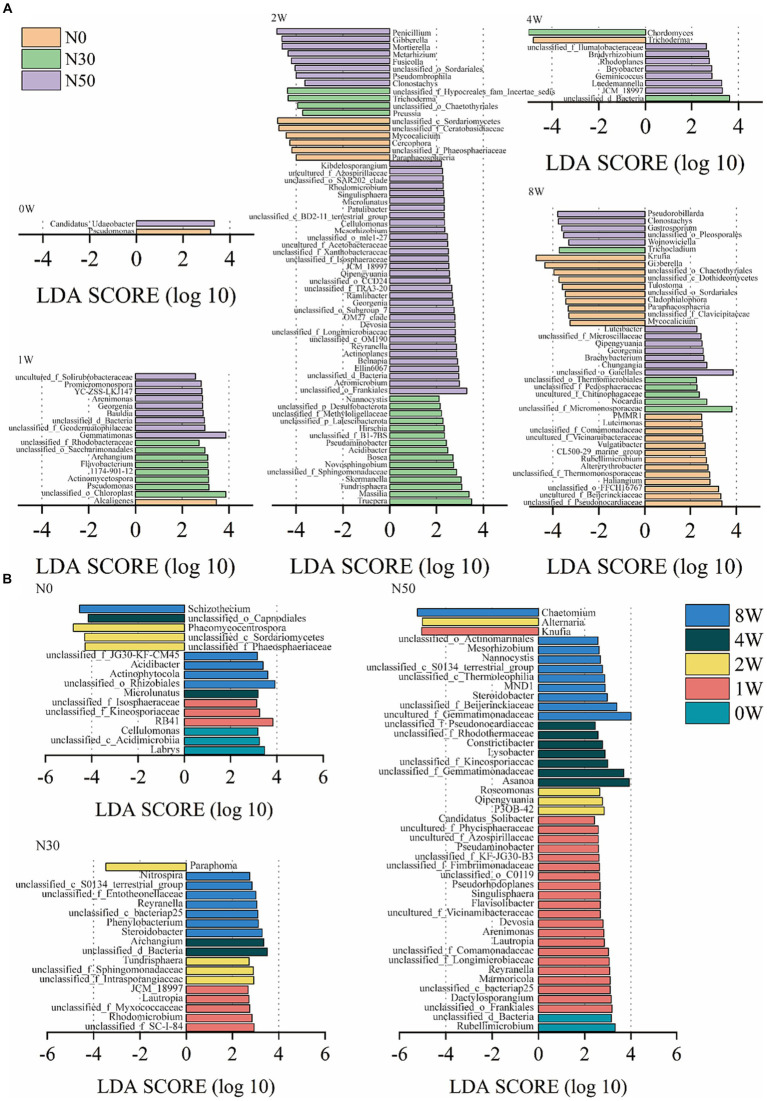
Linear discriminant analysis effect size (LEfSe) analysis showed differentially abundant genera between heavy fractions from the ^13^C-litter decomposition experiment (*p* < 0.05 and LDA score >2.0). The negative *x*-axis represents the fungal community, and its LDA score is regarded as its absolute value. The positive *x*-axis corresponds to the bacterial community. **(A)** LDA of N treatments. **(B)** LDA of decomposition time. N0, control treatment; N30, N addition treatment (30 kg ha^−1^ year^−1^); N50, N addition treatment (50 kg ha^−1^ year^−1^); ^12^C, control (litter decomposition at 0 weeks); ^13^C, ^13^C-litter; 1 W, 2 W, 4 W, and 8 W, litter decomposition at 1, 2, 4, and 8 weeks, respectively.

Additionally, linear discriminant analysis effect size (LEfSe) analysis identified 122 genera with significantly different abundances among the N0, N30, and N50 treatments (*p* < 0.05 and LDA score > 2.0, Figure 5A). Only two genera exhibited significantly different abundances among N0, N30, and N50 treatments at 0 W. However, at 1 W of ^13^C-litter decomposition, compared with the N0 treatment, there were more species with significant differences in N30 and N50 treatments, mainly from *Actinomycetota* and *Pseudomonadota* (Supplementary Table S1). Specifically, *Georgenia*, unclassified_f_*Geodermatophilaceae*, and *Promicromonospora* were significantly enriched in the N50 treatment. At 2 W of decomposition, the presence of fungi differed significantly between N treatments in terms of species, which were mainly from the *Ascomycota*. The N30 treatment primarily involved Trichoderma, *Preussia*, and unclassified_o__*Chaetothyriales*, whereas the N50 treatment mainly comprised Penicillium, *Gibberella*, and unclassified_o__*Sordariales* (Supplementary Table S1). At 2 W of decomposition, bacteria with significantly increased relative abundances in the N30 and N50 treatments compared with the N0 treatment were mainly in the *Actinomycetota* and *Pseudomonadota* phyla. The N30 treatment primarily involved *Massilia*, *Skermanella*, and *Acidibacter*, whereas the N50 treatment mainly comprised unclassified_o__*Frankiales*, *Devosia*, and Ellin6067. JCM_18997 within the *Actinomycetota* was significantly more abundant in the N50 treatment than in the other two treatments at 2 W and 4 W of litter decomposition. At 8 W of ^13^C-litter decomposition, *Pseudorobillarda*, *Wojnowiciella*, and *Luteibacter* exhibited the highest abundances in the N50 treatment; they were absent during the initial decomposition phase (0 W) in all N treatments.

LEfSe analysis identified 72 genera with significantly different abundances at 0 W, 1 W, 2 W, 4 W, and 8 W among the three treatments (*p* < 0.05 and LDA score > 2.0, Figure 5B). More microorganisms were involved in litter decomposition with increased N addition, emphasizing the need for N addition to promote litter decomposition in desert steppes. To confirm that N deposition increased the relative abundances of these microorganisms, we conducted metagenomic analysis in the field to verify changes in the relative abundances of these microorganisms and identify their specific roles.

### 3.3 Metagenomes analysis

**Figure 6:**
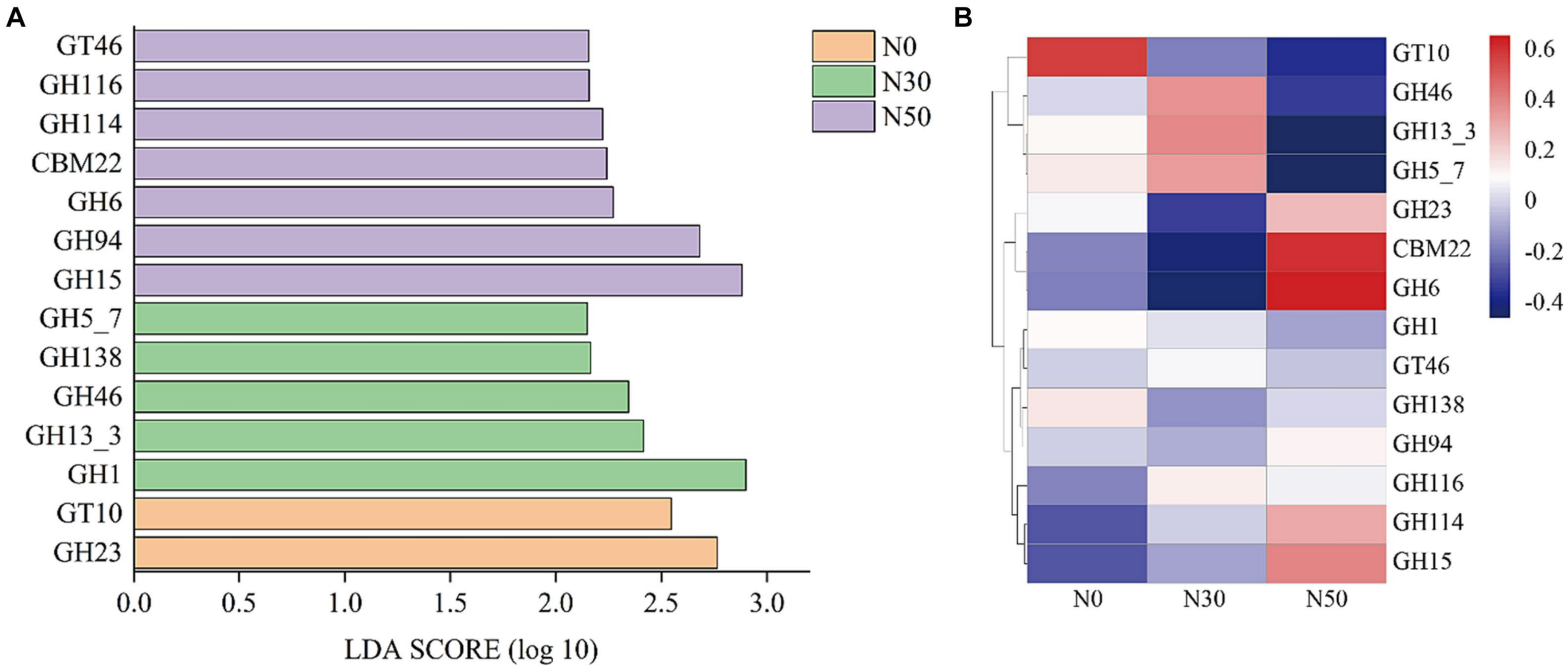
LEfSe screening of significant differences between CAZy gene families (*p* < 0.05 and LDA score >2.0, **A**). The heat maps show the relative abundances (normalized data) of CAZy gene families **(B)**. Family annotations of the genes are based on the carbohydrate-active enzyme database (CAZy). GH: glycoside hydrolase, GT: glycosyltransferase, CBM: carbohydrate-binding module. N0, control treatment; N30, N addition treatment (30 kg ha^−1^ year^−1^); N50, N addition treatment (50 kg ha^−1^ year^−1^).

Metagenomic analysis was performed on each treated soil sample before the DNA-SIP experiment (i.e., at 0 W). Compared with the DNA-SIP experiment, 95 genera were identified in the metagenomic analysis, and the effects of N addition were identical to effects in the DNA-SIP experiment (Supplementary Table S2). The functional genes of 95 genera were selected to establish a subgene set. Because the litter-degrading capacities of microbial consortia are closely related to genes encoding carbohydrate-active enzymes (CAZy), metagenomic data were annotated using the CAZy database. LEfSe analysis identified 14 functional genes with significantly different abundances among the N0, N30, and N50 treatments (*p* < 0.05 and LDA score >2.0) (Figure 6). N30 treatment significantly increased the abundance of genes in the glycoside hydrolase (GH) family (GH13_3, GH46, GH5_7); N50 treatment significantly increased the abundance of genes in the GH family (GH15, GH94, GH6, GH114) and the abundances of genes in the carbohydrate-binding module (CBM, CBM22) and glycoside transferase (GT, GT46) families. GH family genes were mainly derived from *Actinomycetota* and *Pseudomonadota* (Supplementary Table S3). CBM family genes were mainly derived from *Actinomycetota*. GT family genes were mainly derived from *Pseudomonadota*. Thus, N deposition may enhance litter decomposition in desert steppes by promoting the GH pathway.

**Figure 7:**
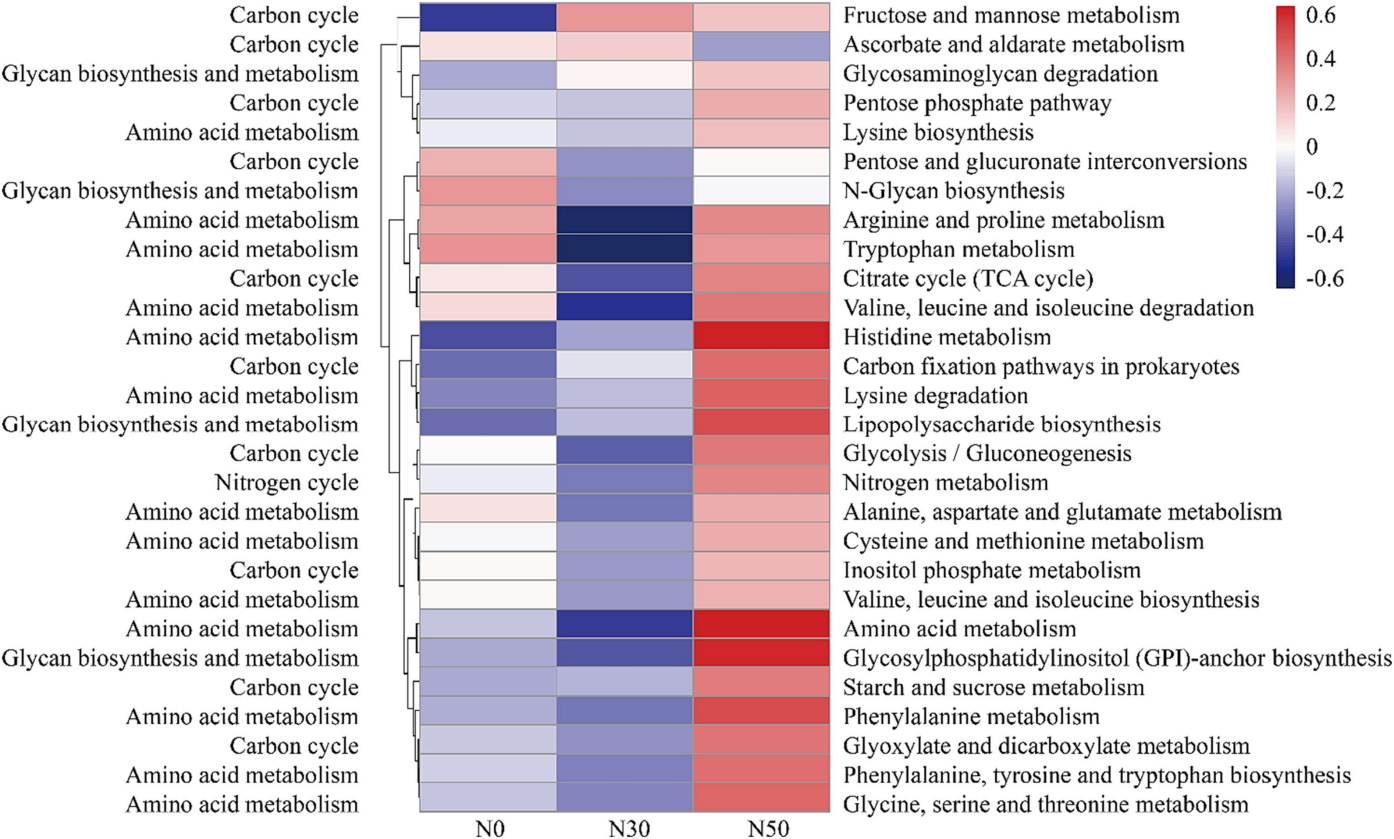
Abundances of C and N cycle-related functional genes based on the KEGG database (normalized data). KEGG, Kyoto Encyclopedia of Genes and Genomes. N0, control treatment; N30, N addition treatment (30 kg ha^−1^ year^−1^); N50, N addition treatment (50 kg ha^−1^ year^−1^).

Compared with N0 and N30 treatments, the N50 treatment also increased the abundances of genes related to the C cycle, such as the pentose phosphate pathway, citrate cycle (tricarboxylic acid cycle), C fixation pathways in prokaryotes, glycolysis (gluconeogenesis), starch and sucrose metabolism, glyoxylate and dicarboxylate metabolism, and inositol phosphate metabolism (Figure 7). Additionally, the data showed higher relative abundances of N cycle genes (N metabolism) in the N50 treatment, compared with the N30 and N0 treatments (Figure 7). The N50 treatment resulted in higher relative abundances of genes related to amino acid metabolism (except tryptophan metabolism) and glycan biosynthesis and metabolism (except N-glycan biosynthesis), compared with the N30 and N0 treatments (Figure 7). These results indicate that continuous N addition stimulates the enrichment of soil microbial genes related to N and C fixation, thereby facilitating litter decomposition and soil C accumulation.

## 4 Discussion

Soil bacteria and fungi participate in the transformation and stabilization of plant litter-derived C (Liang et al., 2017; Guo et al., 2022). In the present study, after 1 W of ^13^C-litter incubation, there were significant differences in bacterial community composition among N treatments (Figure 3). Bacteria may show high affinity to available organic substrates, especially polysaccharides, which decompose first in the early stage of litter decomposition; thus, bacterial abundances differed first among N treatments (Romaní et al., 2006; Müller et al., 2017). From 2 W to 4 W of ^13^C-litter incubation, there were significant differences in fungi community composition among N treatments (Figure 3). Eukaryotic fungal cells are larger and more complex than prokaryotic bacterial cells, with a smaller surface area-to-volume ratio (Moore et al., 2005; Müller et al., 2017). Therefore, a longer duration of measurement is needed to determine ^13^C enrichment and turnover in fungi. Analysis of decomposition at 2 W and 4 W showed that bacterial β-diversity did not significantly differ among N treatments. After 8 W of decomposition, there were significant differences in bacterial and fungal communities among N treatments. The consensus view is that fungi primarily decompose high-molecular and/or lignified compounds, whereas bacteria subsequently decompose polysaccharides and polymeric compounds (Romaní et al., 2006). These processes may explain why there were no differences in the bacterial community at 2 W and 4 W, but a difference was observed at 8 W of decomposition. At the phylum level, *Actinomycetota* and *Ascomycota* were predominantly involved in ^13^C-litter utilization in desert steppes (Figure 4). *Actinomycetota* can efficiently grow on recalcitrant polymers such as lignocellulose (Adhi et al., 1989). They also have stronger decomposition ability in soils with relatively poor nutrients because their functional genes promote decomposition and more prominent interspecific interactions (Adhi et al., 1989; Bao et al., 2021). In most previous studies, fungi from the *Ascomycota* and a few members of *Basidiomycota* were dominant in the early stages of litter decomposition (Zheng et al., 2021). The abundance of *Ascomycota* fungi gradually decreases during decomposition because they are gradually replaced by *Basidiomycota* fungi, especially saprotrophic cord formers (Voříšková and Baldrian, 2013). This conclusion is universal among desert steppes. Notably, *Actinomycetota* (relative abundance 51.21–61.09%) and *Ascomycota* (relative abundance 65.95–80.91%) were most abundant at 0 W, implying that most *Actinomycetota* and *Ascomycota* microorganisms are not involved in litter decomposition.

At 2 W of ^13^C-litter decomposition, the relative abundances of *Gibberella*, *Mortierella*, and *Penicillium* were significantly higher in the N50 treatment than in the N0 and N30 treatments (Figure 5A). *Mortierella* and *Penicillium* are associated with N cycling (Chen et al., 2023). In the N50 treatment, the relative abundance of *Knufia* was significantly higher at 1 W compared with the other decomposition time points, whereas the relative abundance of *Chaetomium* was significantly higher at 8 W compared with other time points. *Chaetomium* has been confirmed as an important participant in organic matter decomposition and a major contributor of microbial-derived C during litter decomposition (Wang et al., 2023). Although some microorganisms were identified as taxa with significantly increased relative abundances, those relative abundances were also high at 0 W (Supplementary Table S1). Bacteria with these patterns included uncultured_f__*Gemmatimonadaceae*, *Asanoa*, unclassified_o__*Gaiellales*, unclassified_f__JG30-KF-CM45, and *Gemmatimonas*. Fungi with these patterns included *Gibberella*, *Mortierella*, unclassified_c__*Sordariomycetes*, unclassified_f__*Ceratobasidiaceae*, and *Alternaria*. Moreover, these microorganisms (except for *Asanoa* and *Gemmatimonas*) were not detected in subsequent metagenomic analyses. Further research is needed to determine whether these microorganisms are truly involved in the decomposition of desert steppe litter. Importantly, most labeled microorganisms belong to undescribed taxonomic groups; therefore, further research is needed to explore the diversity of litter C microbial utilizers in soils.

Compared with the metagenomic analysis, 95 genera of microorganisms appeared in both analyses (Supplementary Table S2). Several genera, such as *Lysobacter* (Luo et al., 2017), *Cellulomonas*, *Dactylosporangium* (Gao et al., 2022), *Altererythrobacter* (Na et al., 2019) and *Massilia* (Liu et al., 2021), had putative contributions to organic matter degradation in the current study. For example, *Lysobacter*, a member of *Pseudomonadota*, was identified as a common organic matter utilizer and is known to possess strong cellulolytic ability for breaking down biomacromolecules (Luo et al., 2017; Fu et al., 2022). *Massilia* may have the ability to produce chitinase (Liu et al., 2021). Both *Lysobacter* and *Altererythrobacter* are resource-limited populations; their abundances can be increased by environmental improvement (Xu et al., 2018; Na et al., 2019). Some studies have shown that shifts in the relative abundance of *Altererythrobacter* exhibited a unimodal relationship with the rate of N addition (Huang J. et al., 2021). Furthermore, some microorganisms possess cellulose degradation ability, such as *Luteimonas*, *Lysobacter*, and *Promicromonospora*. For example, *Luteimonas* produces cellulase and *hemicellulase*, accelerating cellulose decomposition (He et al., 2022). *Promicromonospora* can decompose cellulose, cellobiose, *xylan*, or lignocellulosic materials in soil (Yang et al., 2022). *Bradyrhizobium* promotes the conversion of non-labile phosphorus (P) to labile P forms through microbial immobilization and subsequent mineralization. Additionally, *Bradyrhizobium* harbors an N-fixing gene, suggesting that this genus could play an important role in coupled N/P cycling in soils with sufficient C (Luo et al., 2017; Huang Y. et al., 2021). The abundances of these microorganisms increase with N addition (Supplementary Table S2), and they carry functional genes in the GH and CBM families (Supplementary Table S3). Microorganisms carrying functional GH family genes can produce carbohydrate enzymes, which facilitate the hydrolysis of glycosidic bonds in complex sugars into carbohydrates. However, our results showed that N addition significantly increased genes in the GH family (GH15, GH94, GH6, GH114) and genes in the CBM (CBM, CBM22) and GT (GT, GT46) families (Figure 6B). This also represents one pathway through which N deposition promotes litter decomposition in desert steppes. A similar conclusion can be derived from the KEGG results, where both C cycle and glycan biosynthesis and metabolism functions were enhanced in the N50 treatment (Figure 7).

Several genera, such as unclassified_f__*Gemmatimonadaceae*, *Microlunatus*, *Mesorhizobium*, *Steroidobacter*, *Nitrospira*, *Georgenia*, *Ramlibacter*, *Brachybacterium*, and *Arenimonas*, had putative contributions to the N cycle. The abundances of genes related to N cycling and amino acid metabolism increased in the N50 treatment (Figure 7). These increases mainly occurred because of increased abundances of these microorganisms, which also provide a source of N for litter decomposition in desert steppes. Desert steppe soils contain diverse N cycles bacteria such as *Gemmatimonadaceae* (Ni et al., 2023), *Mesorhizobium* (Zhang et al., 2023), *Steroidobacter* (Zhao et al., 2022), *Georgenia* (Zhou et al., 2019), *Arenimonas* (Zhang et al., 2020). They are also the major group involved in the decomposition of desert steppe litter, and they carry GH family genes (e.g., GH6, GH15, GH23, GH94, and GH114). Notably, N deposition increased the abundances of these microorganisms (Supplementary Table S2), which is the primary reason for the highest abundance of GH family genes in the N50 treatment (Figure 6B). This result is consistent with previous research findings that N deposition can increase the abundances of N cycles bacteria in desert steppe soils (Zhou et al., 2019). N cycles bacteria must consume large amounts of C during nitrification and denitrification processes, which may explain why N deposition promotes litter decomposition in desert steppes.

## 5 Conclusion

In this study, the effects of N deposition on microbial species involved in litter decomposition in desert steppes were investigated using DNA-SIP in combination with high-throughput sequencing techniques. Metagenomic technology was used to conduct a preliminary exploration of microbial mechanisms underlying the impact of N deposition on litter decomposition in desert steppes. The current study showed that: (1) *Actinomycetota*, *Pseudomonadota*, and *Ascomycota* are mainly involved in litter decomposition in desert steppes; (2) N deposition (50 kg ha^−1^ year^−1^) significantly increased the relative abundance of some microorganisms involved in the decomposition process; and (3) N deposition may promote litter decomposition in desert steppes by increasing the abundances of N cycles bacteria (usually carrying GH family functional genes). These findings contribute to a deeper understanding of the C assimilation mechanisms associated with litter residue production, emphasizing the importance of extensive C utilization.

## Data availability statement

The datasets presented in this study can be found in online repositories. The names of the repository/repositories and accession number(s) can be found below: PRJNA1015017 and PRJNA979949.

## Author contributions

HY: Data curation, Formal analysis, Writing – original draft. NT: Data curation, Writing – original draft. ZW: Data curation, Writing – original draft. SH: Data curation, Writing – original draft. YZ: Formal analysis, Writing – original draft. MY: Formal analysis, Writing – original draft. MH: Conceptualization, Funding acquisition, Writing – review & editing.
