# Supplementary material for: Identification of bacteria and fungi responsible for litter decomposition in desert steppes via combined DNA stable isotope probing
Source: Front Microbiol. 2024 Mar 8;15:1353629. doi: 10.3389/fmicb.2024.1353629 (PMC10957780; doi:10.3389/fmicb.2024.1353629)
Supplement: Supplementary file 1 [file Data_Sheet_1.docx]

Supplementary Material

# Supplementary Figures and Tables

## Supplementary Figures


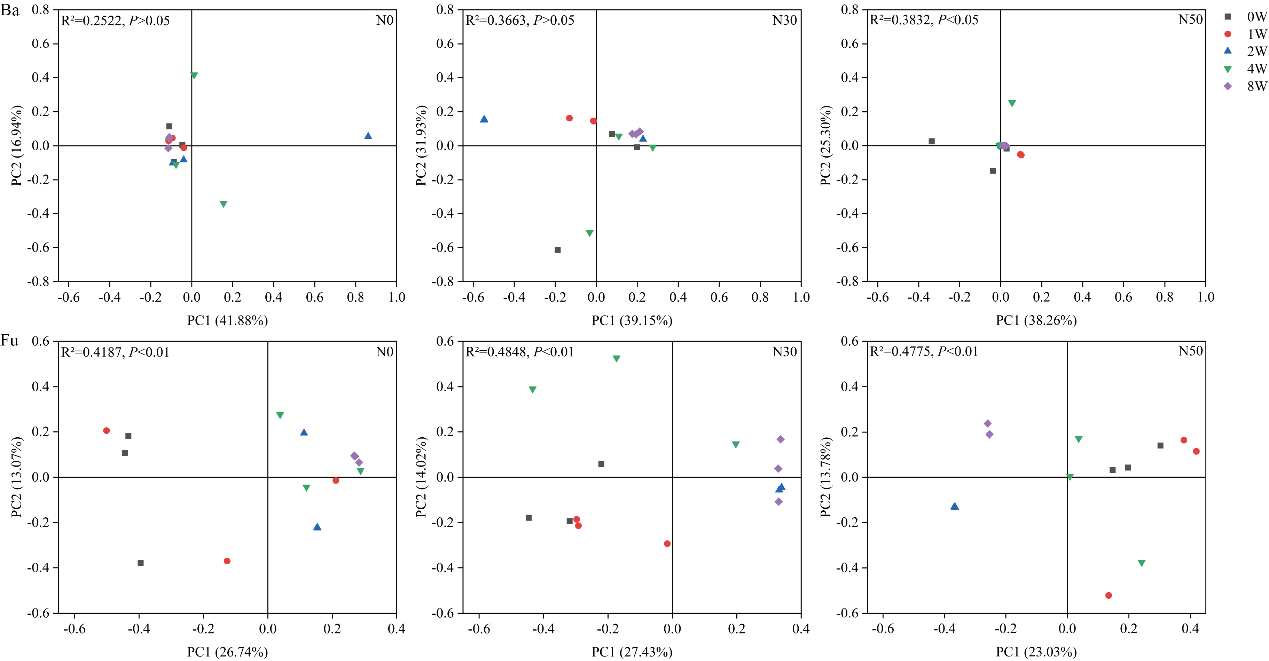


Supplementary Figure 1. PCoA analyses of ^13^C-labeled bacterial communities (Ba) and fungal communities (Fu) involved in decomposition of litter based on OTU level by abund-jaccard distances. N0, control treatment; N30, N addition treatment (30 kg N ha^-1^ yr^-1^); N50, N addition treatment (50 kg N ha^-1^ yr^-1^). ^12^C, control (litter decomposition 0 week); ^13^C, ^13^C-litter; 1W, litter decomposition 1 week; 2W, litter decomposition 2 week; 4W, litter decomposition 4 week; 8W, litter decomposition 8 week. (n = 3).


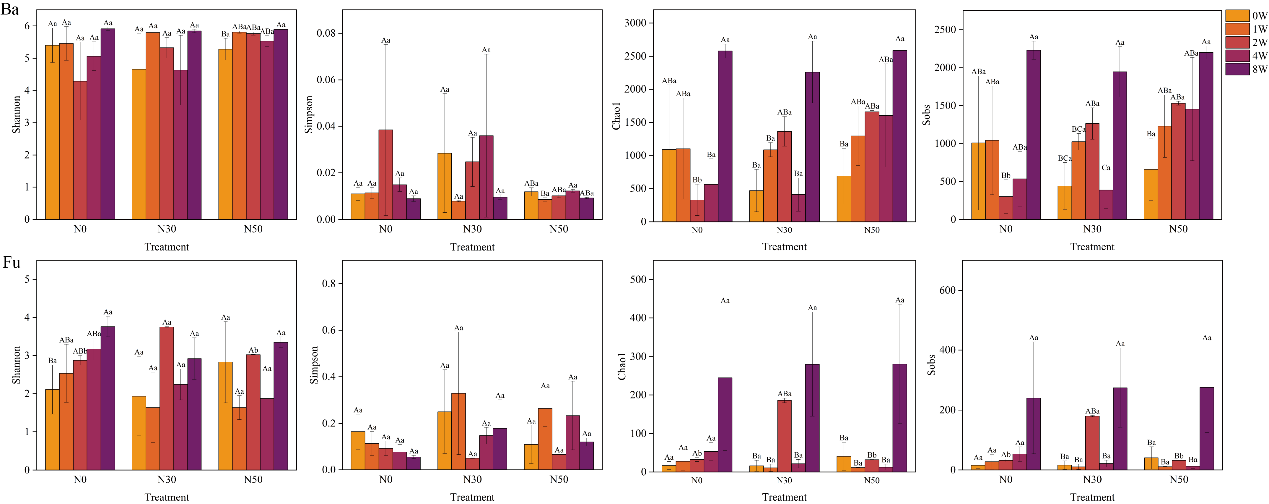


**Supplementary Figure 2.** α-diversity of bacterial communities (Ba) and fungal communities (Fu) based on OTU level. Capital and lowercase letters indicate significant differences among time (0W, 1W, 2W, 4W, 8W) for the same N treatments and among N treatments for the same time, respectively, at *P* < 0.05. N0, control treatment; N30, N addition treatment (30 kg N ha^-1^ yr^-1^); N50, N addition treatment (50 kg N ha^-1^ yr^-1^). ^12^C, control (litter decomposition 0 week); ^13^C, ^13^C-litter; 1W, litter decomposition 1 week; 2W, litter decomposition 2 week; 4W, litter decomposition 4 week; 8W, litter decomposition 8 week. (n = 3)

## Supplementary Tables

**Supplementary Table 1.** Relative abundance of microorganisms screened by Linear discriminant analysis effect size (LEfSe)

| Type | Phylum | Genus | N0 | | | | | N30 | | | | | N50 | | | | |
| --- | --- | --- | --- | --- | --- | --- | --- | --- | --- | --- | --- | --- | --- | --- | --- | --- | --- |
|  |  |  | 0W | 1W | 2W | 4W | 8W | 0W | 1W | 2W | 4W | 8W | 0W | 1W | 2W | 4W | 8W |
| Fungi | Ascomycota | Penicillium | 8.372 | 5.470 | 2.995 | 15.361 | 6.690 | 6.647 | 4.376 | 13.047 | 9.007 | 9.459 | 18.241 | 21.087 | 17.066 | 28.820 | 11.885 |
| Fungi | Ascomycota | Chaetomium | 11.130 | 1.579 | 5.671 | 4.817 | 14.184 | 2.607 | 0.000 | 12.927 | 17.562 | 30.950 | 12.241 | 0.000 | 19.511 | 12.416 | 32.259 |
| Fungi | Ascomycota | Knufia | 0.000 | 12.939 | 11.883 | 10.616 | 14.968 | 0.000 | 7.408 | 7.556 | 3.745 | 3.881 | 2.492 | 21.236 | 7.271 | 0.000 | 8.421 |
| Fungi | Ascomycota | Gibberella | 6.742 | 1.027 | 2.429 | 2.742 | 6.951 | 1.115 | 1.310 | 6.980 | 3.015 | 2.438 | 1.021 | 0.000 | 10.652 | 0.000 | 3.456 |
| Fungi | Ascomycota | unclassified_c__Sordariomycetes | 0.002 | 0.765 | 13.034 | 3.728 | 2.839 | 2.675 | 2.516 | 2.238 | 0.000 | 1.561 | 0.817 | 2.574 | 0.000 | 0.000 | 1.239 |
| Fungi | Ascomycota | Alternaria | 8.334 | 0.162 | 4.929 | 1.260 | 0.391 | 0.000 | 0.001 | 1.418 | 3.112 | 0.195 | 1.641 | 0.000 | 4.453 | 0.283 | 0.409 |
| Fungi | Ascomycota | Trichoderma | 0.000 | 4.627 | 1.669 | 7.361 | 1.659 | 0.000 | 2.873 | 4.169 | 0.552 | 0.844 | 0.342 | 0.000 | 0.000 | 0.000 | 1.815 |
| Fungi | Ascomycota | Schizothecium | 0.000 | 0.202 | 0.310 | 3.696 | 5.762 | 0.000 | 0.000 | 0.727 | 0.291 | 7.375 | 0.180 | 0.000 | 0.000 | 0.977 | 5.170 |
| Fungi | Ascomycota | Chordomyces | 4.809 | 0.000 | 0.000 | 0.000 | 0.000 | 0.000 | 0.000 | 0.000 | 13.607 | 0.000 | 0.000 | 0.000 | 1.610 | 0.001 | 0.002 |
| Fungi | Ascomycota | Phaeomycocentrospora | 0.000 | 0.108 | 3.262 | 0.958 | 0.622 | 0.000 | 0.000 | 2.480 | 0.517 | 0.494 | 5.212 | 0.000 | 3.218 | 0.000 | 0.919 |
| Fungi | Ascomycota | unclassified_o__Sordariales | 0.000 | 0.000 | 0.000 | 0.000 | 0.632 | 0.000 | 10.002 | 0.288 | 0.000 | 0.220 | 2.501 | 0.000 | 1.041 | 2.412 | 0.075 |
| Fungi | Ascomycota | unclassified_c__Dothideomycetes | 0.000 | 2.032 | 5.038 | 1.737 | 1.249 | 0.000 | 0.000 | 0.440 | 0.000 | 0.149 | 2.459 | 0.000 | 3.368 | 0.042 | 0.633 |
| Fungi | Ascomycota | Preussia | 0.125 | 0.241 | 0.000 | 0.832 | 0.669 | 8.662 | 0.000 | 0.830 | 0.000 | 1.008 | 0.518 | 0.000 | 0.648 | 0.002 | 0.412 |
| Fungi | Ascomycota | Cercophora | 0.000 | 0.872 | 3.456 | 3.436 | 2.680 | 0.000 | 0.000 | 0.585 | 0.000 | 1.778 | 0.000 | 0.000 | 0.000 | 0.000 | 1.124 |
| Fungi | Ascomycota | unclassified_o__Chaetothyriales | 0.000 | 0.460 | 1.052 | 2.844 | 2.399 | 0.000 | 0.000 | 1.754 | 0.000 | 0.601 | 0.000 | 0.000 | 0.000 | 0.000 | 1.512 |
| Fungi | Ascomycota | unclassified_o__Capnodiales | 0.000 | 0.313 | 0.244 | 1.492 | 0.885 | 0.000 | 0.000 | 0.079 | 0.759 | 0.239 | 0.000 | 0.000 | 3.986 | 1.662 | 0.901 |
| Fungi | Ascomycota | Pseudorobillarda | 0.000 | 0.000 | 0.000 | 0.000 | 0.000 | 0.000 | 0.000 | 0.000 | 0.000 | 0.008 | 0.000 | 5.811 | 0.000 | 0.000 | 1.220 |
| Fungi | Ascomycota | unclassified_f__Phaeosphaeriaceae | 0.000 | 0.251 | 2.797 | 0.250 | 0.553 | 0.000 | 0.000 | 0.473 | 0.000 | 0.627 | 0.189 | 0.007 | 0.000 | 0.000 | 0.486 |
| Fungi | Ascomycota | unclassified_o__Pleosporales | 0.002 | 1.466 | 0.000 | 0.013 | 0.468 | 0.000 | 0.000 | 1.322 | 0.411 | 0.102 | 0.305 | 0.000 | 0.000 | 0.000 | 0.740 |
| Fungi | Ascomycota | Clonostachys | 0.000 | 0.000 | 0.229 | 0.644 | 0.305 | 0.000 | 0.000 | 0.769 | 0.000 | 0.017 | 0.167 | 0.000 | 0.953 | 0.000 | 1.156 |
| Fungi | Ascomycota | Fusicolla | 0.000 | 0.000 | 0.000 | 1.028 | 0.064 | 0.000 | 0.000 | 0.428 | 0.000 | 0.405 | 0.000 | 0.000 | 1.454 | 0.000 | 0.289 |
| Fungi | Ascomycota | Cladophialophora | 0.000 | 0.000 | 0.000 | 0.000 | 0.356 | 0.000 | 0.000 | 0.054 | 0.000 | 0.049 | 0.317 | 2.666 | 0.000 | 0.000 | 0.172 |
| Fungi | Ascomycota | Paraphaeosphaeria | 0.000 | 0.538 | 1.903 | 0.000 | 0.364 | 0.000 | 0.000 | 0.391 | 0.000 | 0.059 | 0.000 | 0.000 | 0.000 | 0.000 | 0.017 |
| Fungi | Ascomycota | Pseudombrophila | 0.000 | 0.159 | 0.000 | 0.000 | 0.337 | 0.000 | 0.000 | 0.094 | 0.000 | 0.061 | 0.000 | 0.000 | 0.871 | 0.000 | 1.289 |
| Fungi | Ascomycota | Metarhizium | 0.000 | 0.000 | 0.000 | 0.000 | 0.188 | 1.246 | 0.000 | 0.024 | 0.000 | 0.107 | 0.000 | 0.000 | 1.062 | 0.000 | 0.070 |
| Fungi | Ascomycota | Paraphoma | 0.000 | 0.000 | 0.000 | 0.933 | 0.383 | 0.112 | 0.001 | 0.288 | 0.000 | 0.081 | 0.000 | 0.000 | 0.000 | 0.000 | 0.210 |
| Fungi | Ascomycota | unclassified_f__Hypocreales_fam_Incertae_sedis | 0.000 | 0.000 | 0.001 | 0.060 | 0.017 | 0.000 | 0.000 | 0.103 | 0.000 | 0.014 | 0.000 | 0.000 | 0.000 | 0.000 | 0.117 |
| Fungi | Ascomycota | Trichocladium | 0.000 | 0.000 | 0.000 | 0.000 | 0.000 | 0.000 | 0.000 | 0.000 | 0.000 | 0.025 | 0.201 | 0.000 | 0.000 | 0.000 | 0.001 |
| Fungi | Ascomycota | Myrmecridium | 0.000 | 0.000 | 0.000 | 0.000 | 0.038 | 0.000 | 0.000 | 0.000 | 0.000 | 0.005 | 0.000 | 0.000 | 0.000 | 0.000 | 0.022 |
| Fungi | Ascomycota | Wojnowiciella | 0.000 | 0.000 | 0.000 | 0.000 | 0.000 | 0.000 | 0.000 | 0.000 | 0.000 | 0.002 | 0.000 | 0.000 | 0.000 | 0.000 | 0.058 |
| Fungi | Ascomycota | unclassified_f__Clavicipitaceae | 0.000 | 0.000 | 0.000 | 0.000 | 0.052 | 0.000 | 0.000 | 0.000 | 0.000 | 0.006 | 0.000 | 0.000 | 0.000 | 0.000 | 0.002 |
| Bacteria | Actinomycetota | Asanoa | 3.460 | 3.434 | 2.493 | 2.800 | 2.623 | 2.632 | 3.002 | 1.374 | 2.110 | 2.118 | 2.536 | 3.362 | 2.149 | 3.711 | 2.826 |
| Bacteria | Actinomycetota | unclassified_o__Gaiellales | 1.451 | 1.898 | 2.325 | 1.653 | 1.922 | 2.172 | 2.312 | 1.226 | 1.618 | 2.702 | 4.119 | 2.382 | 3.649 | 2.614 | 3.125 |
| Bacteria | Actinomycetota | unclassified_f__Micromonosporaceae | 0.516 | 0.880 | 0.893 | 1.090 | 1.130 | 0.730 | 1.083 | 0.327 | 0.617 | 2.467 | 1.326 | 1.585 | 1.108 | 1.633 | 1.445 |
| Bacteria | Actinomycetota | unclassified_o__Frankiales | 0.770 | 0.711 | 0.389 | 0.627 | 0.383 | 0.309 | 0.422 | 0.326 | 0.761 | 0.420 | 0.610 | 0.569 | 0.596 | 0.611 | 0.432 |
| Bacteria | Actinomycetota | unclassified_c__Acidimicrobiia | 0.620 | 0.289 | 0.147 | 0.124 | 0.256 | 0.337 | 0.347 | 0.150 | 0.361 | 0.224 | 0.432 | 0.360 | 0.323 | 0.281 | 0.235 |
| Bacteria | Actinomycetota | Marmoricola | 0.344 | 0.215 | 0.319 | 0.151 | 0.191 | 0.364 | 0.449 | 0.234 | 0.122 | 0.247 | 0.146 | 0.399 | 0.338 | 0.387 | 0.243 |
| Bacteria | Actinomycetota | Actinophytocola | 0.231 | 0.326 | 0.126 | 0.091 | 0.510 | 0.240 | 0.208 | 0.130 | 0.056 | 0.281 | 0.386 | 0.270 | 0.236 | 0.320 | 0.371 |
| Bacteria | Actinomycetota | unclassified_f__Kineosporiaceae | 0.186 | 0.326 | 0.100 | 0.028 | 0.261 | 0.298 | 0.251 | 0.247 | 0.217 | 0.200 | 0.140 | 0.312 | 0.333 | 0.358 | 0.234 |
| Bacteria | Actinomycetota | Luedemannella | 0.128 | 0.273 | 0.147 | 0.191 | 0.168 | 0.284 | 0.360 | 0.100 | 0.075 | 0.207 | 0.267 | 0.171 | 0.172 | 0.404 | 0.252 |
| Bacteria | Actinomycetota | CL500-29_marine_group | 0.148 | 0.195 | 0.333 | 0.121 | 0.228 | 0.287 | 0.269 | 0.101 | 0.134 | 0.141 | 0.278 | 0.173 | 0.185 | 0.116 | 0.167 |
| Bacteria | Actinomycetota | Dactylosporangium | 0.133 | 0.308 | 0.127 | 0.186 | 0.211 | 0.211 | 0.202 | 0.110 | 0.223 | 0.171 | 0.022 | 0.300 | 0.140 | 0.272 | 0.191 |
| Bacteria | Actinomycetota | unclassified_c__Thermoleophilia | 0.085 | 0.116 | 0.056 | 0.684 | 0.162 | 0.237 | 0.205 | 0.081 | 0.114 | 0.168 | 0.063 | 0.179 | 0.174 | 0.126 | 0.186 |
| Bacteria | Actinomycetota | Actinomycetospora | 0.123 | 0.039 | 0.211 | 0.097 | 0.123 | 0.200 | 0.307 | 0.150 | 0.312 | 0.067 | 0.241 | 0.239 | 0.197 | 0.155 | 0.174 |
| Bacteria | Actinomycetota | Aeromicrobium | 0.219 | 0.075 | 0.016 | 0.033 | 0.118 | 0.043 | 0.083 | 0.057 | 0.134 | 0.111 | 0.097 | 0.146 | 0.183 | 0.161 | 0.168 |
| Bacteria | Actinomycetota | unclassified_f__Geodermatophilaceae | 0.003 | 0.057 | 0.006 | 0.013 | 0.022 | 0.003 | 0.003 | 0.010 | 0.102 | 0.037 | 0.281 | 0.171 | 0.141 | 0.246 | 0.131 |
| Bacteria | Actinomycetota | unclassified_f__Thermomonosporaceae | 0.233 | 0.094 | 0.003 | 0.116 | 0.170 | 0.001 | 0.034 | 0.163 | 0.066 | 0.094 | 0.003 | 0.032 | 0.055 | 0.038 | 0.039 |
| Bacteria | Actinomycetota | Actinoplanes | 0.022 | 0.016 | 0.001 | 0.039 | 0.035 | 0.134 | 0.117 | 0.032 | 0.048 | 0.042 | 0.160 | 0.087 | 0.149 | 0.117 | 0.134 |
| Bacteria | Actinomycetota | unclassified_f__Intrasporangiaceae | 0.035 | 0.020 | 0.000 | 0.051 | 0.062 | 0.001 | 0.054 | 0.147 | 0.011 | 0.038 | 0.140 | 0.084 | 0.126 | 0.180 | 0.122 |
| Bacteria | Actinomycetota | unclassified_f__Pseudonocardiaceae | 0.010 | 0.031 | 0.148 | 0.002 | 0.480 | 0.017 | 0.039 | 0.013 | 0.078 | 0.077 | 0.009 | 0.025 | 0.046 | 0.048 | 0.032 |
| Bacteria | Actinomycetota | Cellulomonas | 0.154 | 0.080 | 0.000 | 0.007 | 0.040 | 0.105 | 0.100 | 0.017 | 0.000 | 0.026 | 0.019 | 0.031 | 0.040 | 0.020 | 0.023 |
| Bacteria | Actinomycetota | Georgenia | 0.006 | 0.019 | 0.000 | 0.018 | 0.020 | 0.000 | 0.000 | 0.003 | 0.047 | 0.003 | 0.163 | 0.150 | 0.091 | 0.052 | 0.066 |
| Bacteria | Actinomycetota | JCM_18997 | 0.081 | 0.010 | 0.000 | 0.010 | 0.013 | 0.001 | 0.069 | 0.041 | 0.000 | 0.015 | 0.121 | 0.008 | 0.056 | 0.092 | 0.044 |
| Bacteria | Actinomycetota | uncultured_f__Solirubrobacteraceae | 0.001 | 0.000 | 0.000 | 0.040 | 0.010 | 0.001 | 0.001 | 0.014 | 0.110 | 0.006 | 0.115 | 0.019 | 0.018 | 0.006 | 0.012 |
| Bacteria | Actinomycetota | unclassified_o__Actinomarinales | 0.006 | 0.011 | 0.000 | 0.000 | 0.027 | 0.072 | 0.022 | 0.002 | 0.059 | 0.027 | 0.002 | 0.019 | 0.003 | 0.005 | 0.025 |
| Bacteria | Actinomycetota | Promicromonospora | 0.010 | 0.011 | 0.004 | 0.016 | 0.053 | 0.001 | 0.000 | 0.003 | 0.000 | 0.015 | 0.000 | 0.119 | 0.000 | 0.015 | 0.014 |
| Bacteria | Actinomycetota | Microlunatus | 0.002 | 0.001 | 0.000 | 0.038 | 0.010 | 0.005 | 0.011 | 0.005 | 0.007 | 0.011 | 0.028 | 0.011 | 0.026 | 0.021 | 0.012 |
| Bacteria | Actinomycetota | unclassified_f__Ilumatobacteraceae | 0.003 | 0.019 | 0.012 | 0.001 | 0.006 | 0.000 | 0.001 | 0.001 | 0.000 | 0.016 | 0.014 | 0.017 | 0.016 | 0.058 | 0.015 |
| Bacteria | Actinomycetota | Nocardia | 0.010 | 0.001 | 0.000 | 0.000 | 0.014 | 0.000 | 0.000 | 0.000 | 0.000 | 0.093 | 0.000 | 0.003 | 0.004 | 0.011 | 0.009 |
| Bacteria | Actinomycetota | Brachybacterium | 0.000 | 0.001 | 0.000 | 0.068 | 0.000 | 0.000 | 0.000 | 0.064 | 0.000 | 0.001 | 0.000 | 0.000 | 0.000 | 0.000 | 0.006 |
| Bacteria | Actinomycetota | Kibdelosporangium | 0.004 | 0.003 | 0.000 | 0.008 | 0.010 | 0.000 | 0.012 | 0.001 | 0.008 | 0.004 | 0.010 | 0.001 | 0.007 | 0.003 | 0.004 |
| Bacteria | Actinomycetota | Patulibacter | 0.000 | 0.001 | 0.000 | 0.003 | 0.003 | 0.000 | 0.000 | 0.001 | 0.000 | 0.000 | 0.000 | 0.003 | 0.009 | 0.006 | 0.012 |
| Bacteria | Armatimonadota | unclassified_f__Fimbriimonadaceae | 0.003 | 0.010 | 0.000 | 0.000 | 0.004 | 0.000 | 0.000 | 0.016 | 0.000 | 0.007 | 0.000 | 0.029 | 0.008 | 0.001 | 0.002 |
| Bacteria | Acidobacteriota | RB41 | 0.934 | 1.275 | 0.103 | 0.235 | 0.776 | 0.311 | 0.957 | 0.165 | 0.279 | 0.665 | 0.261 | 0.397 | 0.281 | 0.144 | 0.394 |
| Bacteria | Acidobacteriota | unclassified_o__Subgroup_7 | 0.155 | 0.089 | 0.000 | 0.126 | 0.177 | 0.069 | 0.190 | 0.047 | 0.048 | 0.151 | 0.072 | 0.160 | 0.106 | 0.089 | 0.130 |
| Bacteria | Acidobacteriota | Bryobacter | 0.042 | 0.162 | 0.140 | 0.001 | 0.216 | 0.000 | 0.126 | 0.021 | 0.000 | 0.145 | 0.215 | 0.193 | 0.030 | 0.058 | 0.110 |
| Bacteria | Acidobacteriota | uncultured_f__Vicinamibacteraceae | 0.055 | 0.124 | 0.057 | 0.101 | 0.125 | 0.000 | 0.104 | 0.052 | 0.000 | 0.086 | 0.005 | 0.094 | 0.023 | 0.023 | 0.053 |
| Bacteria | Acidobacteriota | Candidatus_Solibacter | 0.008 | 0.007 | 0.000 | 0.000 | 0.034 | 0.000 | 0.000 | 0.000 | 0.000 | 0.029 | 0.000 | 0.036 | 0.006 | 0.001 | 0.023 |
| Bacteria | Bacteroidota | Flavobacterium | 0.009 | 0.005 | 0.429 | 0.638 | 0.006 | 0.017 | 0.210 | 2.100 | 0.000 | 0.000 | 0.000 | 0.000 | 0.014 | 0.000 | 0.000 |
| Bacteria | Bacteroidota | unclassified_f__Rhodothermaceae | 0.212 | 0.005 | 0.017 | 0.000 | 0.025 | 0.000 | 0.018 | 0.016 | 0.000 | 0.015 | 0.002 | 0.001 | 0.025 | 0.057 | 0.022 |
| Bacteria | Bacteroidota | unclassified_f__Sphingobacteriaceae | 0.000 | 0.000 | 0.000 | 0.043 | 0.000 | 0.000 | 0.000 | 0.034 | 0.000 | 0.000 | 0.000 | 0.000 | 0.000 | 0.000 | 0.000 |
| Bacteria | Bacteroidota | uncultured_f__Chitinophagaceae | 0.000 | 0.002 | 0.000 | 0.000 | 0.001 | 0.000 | 0.000 | 0.000 | 0.000 | 0.021 | 0.000 | 0.001 | 0.017 | 0.000 | 0.006 |
| Bacteria | Bacteroidota | Flavihumibacter | 0.000 | 0.000 | 0.000 | 0.000 | 0.000 | 0.000 | 0.000 | 0.033 | 0.000 | 0.000 | 0.000 | 0.000 | 0.000 | 0.000 | 0.000 |
| Bacteria | Bacteroidota | unclassified_f__Microscillaceae | 0.000 | 0.000 | 0.000 | 0.007 | 0.002 | 0.000 | 0.000 | 0.000 | 0.000 | 0.001 | 0.000 | 0.000 | 0.003 | 0.000 | 0.007 |
| Fungi | Basidiomycota | unclassified_f__Ceratobasidiaceae | 3.121 | 7.726 | 9.748 | 0.163 | 2.309 | 0.585 | 1.274 | 0.232 | 0.000 | 1.922 | 0.000 | 0.000 | 0.000 | 0.000 | 0.023 |
| Fungi | Basidiomycota | Gastrosporium | 0.000 | 0.000 | 0.000 | 0.000 | 0.197 | 0.000 | 0.000 | 0.059 | 0.000 | 0.011 | 0.249 | 1.955 | 0.000 | 0.000 | 0.738 |
| Fungi | Basidiomycota | Tulostoma | 0.000 | 0.221 | 0.000 | 0.000 | 0.684 | 0.000 | 0.000 | 0.037 | 0.000 | 0.042 | 0.000 | 0.000 | 0.000 | 0.000 | 0.016 |
| Bacteria | Bdellovibrionota | OM27_clade | 0.076 | 0.065 | 0.006 | 0.002 | 0.145 | 0.053 | 0.092 | 0.027 | 0.116 | 0.189 | 0.036 | 0.106 | 0.089 | 0.051 | 0.125 |
| Bacteria | Chloroflexota | unclassified_f__JG30-KF-CM45 | 2.459 | 2.885 | 1.819 | 1.481 | 3.195 | 1.552 | 2.758 | 1.174 | 2.079 | 3.200 | 1.814 | 1.996 | 1.647 | 2.298 | 2.610 |
| Bacteria | Chloroflexota | unclassified_o__C0119 | 0.029 | 0.074 | 0.048 | 0.013 | 0.051 | 0.031 | 0.154 | 0.029 | 0.020 | 0.053 | 0.016 | 0.084 | 0.020 | 0.006 | 0.048 |
| Bacteria | Chloroflexota | unclassified_o__SAR202_clade | 0.003 | 0.011 | 0.000 | 0.000 | 0.019 | 0.000 | 0.036 | 0.008 | 0.000 | 0.023 | 0.018 | 0.010 | 0.025 | 0.002 | 0.012 |
| Bacteria | Chloroflexota | unclassified_o__Thermomicrobiales | 0.003 | 0.008 | 0.000 | 0.000 | 0.001 | 0.000 | 0.004 | 0.005 | 0.006 | 0.021 | 0.015 | 0.018 | 0.008 | 0.003 | 0.007 |
| Bacteria | Cyanobacteria | unclassified_o__Chloroplast | 0.036 | 0.058 | 0.317 | 2.367 | 0.003 | 0.077 | 1.438 | 7.047 | 0.000 | 0.000 | 0.063 | 0.000 | 0.000 | 0.000 | 0.011 |
| Bacteria | Deinococcota | Truepera | 0.007 | 0.540 | 0.000 | 0.109 | 0.009 | 0.000 | 0.178 | 0.630 | 0.000 | 0.017 | 0.000 | 0.000 | 0.002 | 0.001 | 0.003 |
| Bacteria | Desulfobacterota | unclassified_p__Desulfobacterota | 0.002 | 0.005 | 0.000 | 0.046 | 0.005 | 0.000 | 0.000 | 0.027 | 0.000 | 0.004 | 0.000 | 0.001 | 0.001 | 0.001 | 0.006 |
| Bacteria | Entotheonellaeota | unclassified_f__Entotheonellaceae | 0.097 | 0.076 | 0.000 | 0.022 | 0.177 | 0.032 | 0.175 | 0.048 | 0.000 | 0.191 | 0.048 | 0.097 | 0.066 | 0.047 | 0.161 |
| Bacteria | Bacillota | Chungangia | 0.000 | 0.027 | 0.000 | 0.001 | 0.002 | 0.000 | 0.000 | 0.000 | 0.000 | 0.000 | 0.000 | 0.000 | 0.001 | 0.000 | 0.003 |
| Bacteria | Gemmatimonadota | uncultured_f__Gemmatimonadaceae | 2.990 | 2.805 | 1.615 | 3.465 | 4.764 | 2.210 | 3.543 | 1.423 | 3.274 | 4.821 | 2.108 | 3.472 | 3.209 | 2.597 | 3.970 |
| Bacteria | Gemmatimonadota | Gemmatimonas | 1.476 | 1.370 | 1.260 | 1.126 | 0.984 | 1.409 | 1.640 | 0.708 | 1.112 | 1.122 | 2.800 | 2.839 | 3.819 | 3.091 | 2.425 |
| Bacteria | Gemmatimonadota | unclassified_f__Gemmatimonadaceae | 1.469 | 1.635 | 1.974 | 1.203 | 1.359 | 0.922 | 1.902 | 0.958 | 1.663 | 1.417 | 2.271 | 2.338 | 2.675 | 2.721 | 1.689 |
| Bacteria | Gemmatimonadota | YC-ZSS-LKJ147 | 0.185 | 0.063 | 0.169 | 0.118 | 0.060 | 0.171 | 0.039 | 0.034 | 0.144 | 0.096 | 0.152 | 0.136 | 0.151 | 0.111 | 0.136 |
| Bacteria | Gemmatimonadota | unclassified_f__Longimicrobiaceae | 0.114 | 0.105 | 0.001 | 0.016 | 0.073 | 0.087 | 0.144 | 0.087 | 0.005 | 0.057 | 0.028 | 0.268 | 0.122 | 0.052 | 0.080 |
| Bacteria | Gemmatimonadota | unclassified_c__S0134_terrestrial_group | 0.125 | 0.055 | 0.045 | 0.016 | 0.114 | 0.019 | 0.081 | 0.015 | 0.006 | 0.118 | 0.007 | 0.045 | 0.077 | 0.052 | 0.092 |
| Bacteria | Gemmatimonadota | unclassified_c__BD2-11_terrestrial_group | 0.110 | 0.048 | 0.000 | 0.000 | 0.053 | 0.056 | 0.062 | 0.015 | 0.030 | 0.059 | 0.012 | 0.075 | 0.037 | 0.028 | 0.039 |
| Bacteria | Latescibacterota | unclassified_p__Latescibacterota | 0.055 | 0.011 | 0.000 | 0.011 | 0.011 | 0.000 | 0.000 | 0.033 | 0.000 | 0.006 | 0.000 | 0.000 | 0.004 | 0.004 | 0.007 |
| Fungi | Mortierellomycota | Mortierella | 3.411 | 0.830 | 0.000 | 3.059 | 0.617 | 12.014 | 0.000 | 1.610 | 0.000 | 1.001 | 4.057 | 0.000 | 7.767 | 13.315 | 0.634 |
| Bacteria | Myxococcota | Haliangium | 0.463 | 0.319 | 0.390 | 0.715 | 0.506 | 0.402 | 0.461 | 0.175 | 0.420 | 0.415 | 0.236 | 0.513 | 0.255 | 0.187 | 0.364 |
| Bacteria | Myxococcota | Archangium | 0.146 | 0.065 | 0.111 | 0.344 | 0.107 | 0.009 | 0.287 | 0.116 | 0.574 | 0.082 | 0.030 | 0.250 | 0.111 | 0.149 | 0.200 |
| Bacteria | Myxococcota | unclassified_c__bacteriap25 | 0.084 | 0.127 | 0.030 | 0.388 | 0.353 | 0.059 | 0.178 | 0.038 | 0.086 | 0.288 | 0.084 | 0.321 | 0.116 | 0.075 | 0.246 |
| Bacteria | Myxococcota | unclassified_o__mle1-27 | 0.041 | 0.011 | 0.000 | 0.005 | 0.051 | 0.196 | 0.075 | 0.020 | 0.068 | 0.032 | 0.016 | 0.021 | 0.054 | 0.091 | 0.028 |
| Bacteria | Myxococcota | Nannocystis | 0.050 | 0.023 | 0.000 | 0.000 | 0.050 | 0.036 | 0.130 | 0.022 | 0.155 | 0.060 | 0.013 | 0.037 | 0.002 | 0.043 | 0.045 |
| Bacteria | Myxococcota | unclassified_f__Myxococcaceae | 0.049 | 0.023 | 0.010 | 0.058 | 0.039 | 0.001 | 0.066 | 0.007 | 0.000 | 0.027 | 0.073 | 0.021 | 0.029 | 0.016 | 0.048 |
| Bacteria | Myxococcota | P3OB-42 | 0.004 | 0.016 | 0.000 | 0.000 | 0.004 | 0.039 | 0.001 | 0.019 | 0.000 | 0.015 | 0.000 | 0.002 | 0.017 | 0.003 | 0.003 |
| Bacteria | Myxococcota | Vulgatibacter | 0.003 | 0.001 | 0.000 | 0.029 | 0.009 | 0.000 | 0.000 | 0.002 | 0.000 | 0.001 | 0.044 | 0.000 | 0.020 | 0.002 | 0.004 |
| Bacteria | Myxococcota | unclassified_o__FFCH16767 | 0.001 | 0.004 | 0.000 | 0.000 | 0.008 | 0.000 | 0.000 | 0.000 | 0.000 | 0.001 | 0.000 | 0.049 | 0.000 | 0.000 | 0.000 |
| Bacteria | Nitrospirota | Nitrospira | 0.066 | 0.139 | 0.031 | 0.203 | 0.179 | 0.002 | 0.079 | 0.039 | 0.000 | 0.091 | 0.037 | 0.071 | 0.069 | 0.022 | 0.095 |
| Bacteria | Patescibacteria | unclassified_o__Saccharimonadales | 0.000 | 0.000 | 0.000 | 0.150 | 0.003 | 0.000 | 0.175 | 0.450 | 0.000 | 0.000 | 0.000 | 0.002 | 0.000 | 0.000 | 0.000 |
| Bacteria | Planctomycetota | unclassified_c__OM190 | 0.065 | 0.064 | 0.000 | 0.023 | 0.108 | 0.069 | 0.103 | 0.029 | 0.109 | 0.163 | 0.057 | 0.054 | 0.121 | 0.088 | 0.060 |
| Bacteria | Planctomycetota | unclassified_f__Isosphaeraceae | 0.018 | 0.089 | 0.000 | 0.001 | 0.040 | 0.012 | 0.030 | 0.005 | 0.024 | 0.055 | 0.008 | 0.045 | 0.057 | 0.111 | 0.039 |
| Bacteria | Planctomycetota | Tundrisphaera | 0.001 | 0.105 | 0.001 | 0.010 | 0.006 | 0.001 | 0.035 | 0.052 | 0.000 | 0.005 | 0.053 | 0.044 | 0.041 | 0.152 | 0.017 |
| Bacteria | Planctomycetota | Singulisphaera | 0.006 | 0.018 | 0.000 | 0.003 | 0.008 | 0.017 | 0.018 | 0.003 | 0.060 | 0.024 | 0.003 | 0.042 | 0.027 | 0.012 | 0.026 |
| Bacteria | Planctomycetota | uncultured_f__Phycisphaeraceae | 0.004 | 0.010 | 0.000 | 0.002 | 0.010 | 0.011 | 0.039 | 0.002 | 0.010 | 0.009 | 0.000 | 0.023 | 0.005 | 0.002 | 0.005 |
| Bacteria | Pseudomonadota | unclassified_f__Beijerinckiaceae | 1.632 | 1.691 | 1.435 | 2.052 | 2.367 | 1.974 | 1.912 | 0.532 | 0.984 | 2.186 | 0.728 | 1.061 | 0.981 | 0.695 | 1.211 |
| Bacteria | Pseudomonadota | uncultured_f__Beijerinckiaceae | 0.457 | 0.738 | 0.390 | 0.272 | 0.753 | 0.231 | 0.496 | 0.203 | 0.253 | 0.571 | 0.210 | 0.435 | 0.304 | 0.174 | 0.355 |
| Bacteria | Pseudomonadota | unclassified_o__Rhizobiales | 0.633 | 0.375 | 0.074 | 0.109 | 0.837 | 0.315 | 0.499 | 0.160 | 0.304 | 0.675 | 0.431 | 0.359 | 0.303 | 0.162 | 0.532 |
| Bacteria | Pseudomonadota | Pseudomonas | 0.003 | 0.003 | 0.540 | 0.170 | 0.059 | 0.000 | 0.250 | 0.487 | 3.190 | 0.056 | 0.338 | 0.044 | 0.015 | 0.095 | 0.056 |
| Bacteria | Pseudomonadota | Rubellimicrobium | 0.392 | 0.321 | 0.206 | 0.139 | 0.219 | 0.107 | 0.210 | 0.195 | 0.228 | 0.111 | 0.535 | 0.344 | 0.105 | 0.148 | 0.171 |
| Bacteria | Pseudomonadota | unclassified_f__Comamonadaceae | 0.258 | 0.349 | 0.255 | 0.144 | 0.209 | 0.137 | 0.219 | 0.288 | 0.183 | 0.178 | 0.105 | 0.318 | 0.235 | 0.112 | 0.153 |
| Bacteria | Pseudomonadota | Massilia | 0.116 | 0.036 | 0.000 | 0.216 | 0.020 | 1.634 | 0.067 | 0.509 | 0.000 | 0.046 | 0.102 | 0.061 | 0.015 | 0.084 | 0.063 |
| Bacteria | Pseudomonadota | 1174-901-12 | 0.001 | 0.001 | 0.000 | 0.149 | 0.000 | 0.896 | 0.218 | 0.917 | 0.000 | 0.001 | 0.008 | 0.052 | 0.000 | 0.000 | 0.008 |
| Bacteria | Pseudomonadota | Skermanella | 0.182 | 0.243 | 0.006 | 0.009 | 0.407 | 0.033 | 0.247 | 0.234 | 0.094 | 0.256 | 0.068 | 0.058 | 0.079 | 0.026 | 0.149 |
| Bacteria | Pseudomonadota | MND1 | 0.123 | 0.280 | 0.169 | 0.071 | 0.254 | 0.036 | 0.034 | 0.106 | 0.048 | 0.311 | 0.059 | 0.154 | 0.138 | 0.081 | 0.206 |
| Bacteria | Pseudomonadota | Phenylobacterium | 0.127 | 0.239 | 0.215 | 0.056 | 0.127 | 0.007 | 0.063 | 0.108 | 0.001 | 0.159 | 0.272 | 0.236 | 0.134 | 0.145 | 0.152 |
| Bacteria | Pseudomonadota | Devosia | 0.197 | 0.022 | 0.000 | 0.858 | 0.095 | 0.039 | 0.051 | 0.021 | 0.023 | 0.128 | 0.031 | 0.141 | 0.119 | 0.021 | 0.135 |
| Bacteria | Pseudomonadota | Lysobacter | 0.060 | 0.054 | 0.074 | 0.122 | 0.225 | 0.126 | 0.277 | 0.123 | 0.026 | 0.230 | 0.019 | 0.028 | 0.054 | 0.178 | 0.125 |
| Bacteria | Pseudomonadota | Altererythrobacter | 0.169 | 0.153 | 0.074 | 0.042 | 0.218 | 0.051 | 0.078 | 0.045 | 0.058 | 0.127 | 0.092 | 0.200 | 0.152 | 0.099 | 0.123 |
| Bacteria | Pseudomonadota | Ellin6067 | 0.168 | 0.082 | 0.001 | 0.122 | 0.118 | 0.006 | 0.058 | 0.045 | 0.064 | 0.069 | 0.272 | 0.254 | 0.174 | 0.079 | 0.147 |
| Bacteria | Pseudomonadota | Steroidobacter | 0.123 | 0.087 | 0.067 | 0.018 | 0.366 | 0.006 | 0.104 | 0.041 | 0.036 | 0.381 | 0.010 | 0.108 | 0.054 | 0.043 | 0.170 |
| Bacteria | Pseudomonadota | Reyranella | 0.058 | 0.028 | 0.000 | 0.021 | 0.172 | 0.017 | 0.190 | 0.048 | 0.042 | 0.203 | 0.034 | 0.259 | 0.139 | 0.092 | 0.169 |
| Bacteria | Pseudomonadota | unclassified_f__TRA3-20 | 0.180 | 0.170 | 0.004 | 0.087 | 0.206 | 0.029 | 0.139 | 0.028 | 0.062 | 0.155 | 0.023 | 0.049 | 0.090 | 0.071 | 0.102 |
| Bacteria | Pseudomonadota | PMMR1 | 0.251 | 0.051 | 0.013 | 0.139 | 0.096 | 0.114 | 0.031 | 0.063 | 0.038 | 0.076 | 0.050 | 0.057 | 0.059 | 0.097 | 0.041 |
| Bacteria | Pseudomonadota | unclassified_f__SC-I-84 | 0.034 | 0.062 | 0.031 | 0.030 | 0.088 | 0.038 | 0.164 | 0.035 | 0.025 | 0.095 | 0.164 | 0.059 | 0.151 | 0.049 | 0.108 |
| Bacteria | Pseudomonadota | Alcaligenes | 0.000 | 0.561 | 0.000 | 0.164 | 0.007 | 0.218 | 0.000 | 0.000 | 0.140 | 0.000 | 0.000 | 0.009 | 0.000 | 0.002 | 0.004 |
| Bacteria | Pseudomonadota | Labrys | 0.180 | 0.040 | 0.041 | 0.010 | 0.130 | 0.088 | 0.047 | 0.010 | 0.029 | 0.129 | 0.019 | 0.126 | 0.093 | 0.031 | 0.116 |
| Bacteria | Pseudomonadota | uncultured_f__Acetobacteraceae | 0.030 | 0.031 | 0.000 | 0.020 | 0.102 | 0.173 | 0.193 | 0.021 | 0.070 | 0.085 | 0.056 | 0.138 | 0.048 | 0.032 | 0.080 |
| Bacteria | Pseudomonadota | Bradyrhizobium | 0.034 | 0.072 | 0.093 | 0.004 | 0.171 | 0.046 | 0.053 | 0.012 | 0.000 | 0.117 | 0.048 | 0.058 | 0.104 | 0.069 | 0.122 |
| Bacteria | Pseudomonadota | Acidibacter | 0.084 | 0.038 | 0.000 | 0.006 | 0.216 | 0.008 | 0.070 | 0.052 | 0.057 | 0.150 | 0.032 | 0.119 | 0.029 | 0.028 | 0.108 |
| Bacteria | Pseudomonadota | Ramlibacter | 0.116 | 0.034 | 0.000 | 0.016 | 0.082 | 0.090 | 0.068 | 0.042 | 0.000 | 0.061 | 0.114 | 0.057 | 0.073 | 0.047 | 0.074 |
| Bacteria | Pseudomonadota | Novosphingobium | 0.011 | 0.029 | 0.008 | 0.097 | 0.019 | 0.017 | 0.146 | 0.113 | 0.027 | 0.025 | 0.018 | 0.092 | 0.058 | 0.038 | 0.036 |
| Bacteria | Pseudomonadota | Rhodoplanes | 0.029 | 0.034 | 0.013 | 0.009 | 0.094 | 0.000 | 0.056 | 0.012 | 0.000 | 0.103 | 0.067 | 0.114 | 0.037 | 0.069 | 0.085 |
| Bacteria | Pseudomonadota | unclassified_o__CCD24 | 0.027 | 0.053 | 0.000 | 0.003 | 0.101 | 0.032 | 0.063 | 0.034 | 0.000 | 0.052 | 0.019 | 0.084 | 0.075 | 0.082 | 0.062 |
| Bacteria | Pseudomonadota | Roseomonas | 0.009 | 0.012 | 0.140 | 0.009 | 0.007 | 0.014 | 0.046 | 0.015 | 0.357 | 0.002 | 0.005 | 0.025 | 0.027 | 0.003 | 0.011 |
| Bacteria | Pseudomonadota | unclassified_f__Xanthobacteraceae | 0.183 | 0.026 | 0.000 | 0.013 | 0.083 | 0.000 | 0.103 | 0.029 | 0.015 | 0.066 | 0.013 | 0.013 | 0.057 | 0.013 | 0.052 |
| Bacteria | Pseudomonadota | Constrictibacter | 0.022 | 0.045 | 0.000 | 0.011 | 0.070 | 0.106 | 0.050 | 0.021 | 0.007 | 0.077 | 0.004 | 0.009 | 0.028 | 0.133 | 0.053 |
| Bacteria | Pseudomonadota | Belnapia | 0.007 | 0.021 | 0.001 | 0.007 | 0.006 | 0.088 | 0.001 | 0.008 | 0.001 | 0.041 | 0.078 | 0.169 | 0.083 | 0.044 | 0.062 |
| Bacteria | Pseudomonadota | unclassified_f__Rhodobacteraceae | 0.104 | 0.001 | 0.000 | 0.033 | 0.028 | 0.001 | 0.078 | 0.106 | 0.026 | 0.029 | 0.029 | 0.008 | 0.041 | 0.012 | 0.034 |
| Bacteria | Pseudomonadota | Geminicoccus | 0.020 | 0.066 | 0.077 | 0.006 | 0.048 | 0.016 | 0.050 | 0.019 | 0.000 | 0.022 | 0.010 | 0.013 | 0.061 | 0.054 | 0.062 |
| Bacteria | Pseudomonadota | uncultured_f__Azospirillaceae | 0.021 | 0.241 | 0.000 | 0.000 | 0.057 | 0.000 | 0.002 | 0.002 | 0.019 | 0.048 | 0.008 | 0.048 | 0.023 | 0.005 | 0.044 |
| Bacteria | Pseudomonadota | Lautropia | 0.013 | 0.014 | 0.056 | 0.036 | 0.039 | 0.001 | 0.087 | 0.045 | 0.000 | 0.030 | 0.000 | 0.116 | 0.018 | 0.010 | 0.029 |
| Bacteria | Pseudomonadota | Luteimonas | 0.022 | 0.028 | 0.000 | 0.027 | 0.099 | 0.000 | 0.091 | 0.008 | 0.030 | 0.060 | 0.004 | 0.019 | 0.020 | 0.019 | 0.046 |
| Bacteria | Pseudomonadota | Rhodomicrobium | 0.007 | 0.037 | 0.000 | 0.011 | 0.092 | 0.008 | 0.125 | 0.001 | 0.023 | 0.041 | 0.005 | 0.047 | 0.008 | 0.006 | 0.025 |
| Bacteria | Pseudomonadota | unclassified_f__B1-7BS | 0.008 | 0.021 | 0.000 | 0.052 | 0.045 | 0.058 | 0.000 | 0.041 | 0.081 | 0.020 | 0.023 | 0.041 | 0.004 | 0.005 | 0.018 |
| Bacteria | Pseudomonadota | Mesorhizobium | 0.014 | 0.016 | 0.000 | 0.011 | 0.066 | 0.000 | 0.085 | 0.021 | 0.000 | 0.078 | 0.006 | 0.019 | 0.032 | 0.014 | 0.054 |
| Bacteria | Pseudomonadota | unclassified_f__Methyloligellaceae | 0.076 | 0.020 | 0.000 | 0.000 | 0.020 | 0.000 | 0.009 | 0.030 | 0.000 | 0.028 | 0.010 | 0.005 | 0.007 | 0.058 | 0.029 |
| Bacteria | Pseudomonadota | Pseudaminobacter | 0.004 | 0.001 | 0.000 | 0.036 | 0.030 | 0.000 | 0.029 | 0.048 | 0.000 | 0.017 | 0.000 | 0.043 | 0.003 | 0.006 | 0.025 |
| Bacteria | Pseudomonadota | Pseudorhodoplanes | 0.011 | 0.012 | 0.000 | 0.000 | 0.022 | 0.000 | 0.001 | 0.008 | 0.000 | 0.018 | 0.000 | 0.073 | 0.017 | 0.005 | 0.022 |
| Bacteria | Pseudomonadota | Qipengyuania | 0.006 | 0.018 | 0.000 | 0.076 | 0.003 | 0.000 | 0.000 | 0.003 | 0.000 | 0.001 | 0.000 | 0.004 | 0.044 | 0.005 | 0.017 |
| Bacteria | Pseudomonadota | Bauldia | 0.006 | 0.004 | 0.001 | 0.000 | 0.023 | 0.000 | 0.000 | 0.016 | 0.002 | 0.013 | 0.022 | 0.023 | 0.019 | 0.006 | 0.029 |
| Bacteria | Pseudomonadota | Bosea | 0.003 | 0.000 | 0.000 | 0.000 | 0.004 | 0.000 | 0.001 | 0.100 | 0.000 | 0.013 | 0.010 | 0.004 | 0.001 | 0.001 | 0.010 |
| Bacteria | Pseudomonadota | unclassified_f__KF-JG30-B3 | 0.024 | 0.019 | 0.000 | 0.000 | 0.012 | 0.000 | 0.000 | 0.000 | 0.000 | 0.017 | 0.000 | 0.033 | 0.001 | 0.004 | 0.020 |
| Bacteria | Pseudomonadota | Arenimonas | 0.006 | 0.002 | 0.000 | 0.011 | 0.007 | 0.022 | 0.004 | 0.003 | 0.000 | 0.007 | 0.000 | 0.025 | 0.009 | 0.007 | 0.024 |
| Bacteria | Pseudomonadota | Hirschia | 0.011 | 0.000 | 0.000 | 0.000 | 0.012 | 0.000 | 0.000 | 0.044 | 0.000 | 0.017 | 0.000 | 0.000 | 0.019 | 0.003 | 0.003 |
| Bacteria | Pseudomonadota | Luteibacter | 0.000 | 0.000 | 0.000 | 0.000 | 0.003 | 0.000 | 0.000 | 0.000 | 0.000 | 0.000 | 0.000 | 0.000 | 0.000 | 0.002 | 0.031 |
| Bacteria | unclassified_d__Bacteria | unclassified_d__Bacteria | 0.096 | 0.185 | 0.076 | 0.052 | 0.118 | 0.018 | 0.105 | 0.154 | 0.801 | 0.180 | 0.424 | 0.240 | 0.228 | 0.167 | 0.142 |
| Bacteria | Verrucomicrobiota | Candidatus_Udaeobacter | 0.121 | 0.096 | 0.000 | 0.000 | 0.034 | 0.000 | 0.047 | 0.002 | 0.000 | 0.029 | 0.009 | 0.021 | 0.019 | 0.010 | 0.020 |
| Bacteria | Verrucomicrobiota | unclassified_f__Pedosphaeraceae | 0.000 | 0.000 | 0.000 | 0.000 | 0.002 | 0.000 | 0.000 | 0.000 | 0.000 | 0.015 | 0.000 | 0.000 | 0.000 | 0.001 | 0.000 |

The data units in the graph are expressed as %.

Supplementary Table 2. Reads number of microbial filtered by linear discriminant analysis effect size (LEfSe)—metagenomes analysis

| Type | Phylum | Genus | Total reads number | | |
| --- | --- | --- | --- | --- | --- |
|  |  |  | N0 | N30 | N50 |
| Bacteria | Actinomycetota | unclassified_c__Thermoleophilia | 193725 | 183984 | 197484 |
| Bacteria | Actinomycetota | unclassified_c__Acidimicrobiia | 170151 | 167688 | 172435 |
| Bacteria | Actinomycetota | Actinoplanes | 142624 | 145587 | 149518 |
| Bacteria | Actinomycetota | Microlunatus | 130939 | 134431 | 139440 |
| Bacteria | Actinomycetota | Actinophytocola | 49569 | 51152 | 62300 |
| Bacteria | Actinomycetota | Asanoa | 27069 | 26979 | 27407 |
| Bacteria | Actinomycetota | unclassified_f__Micromonosporaceae | 23178 | 23679 | 25800 |
| Bacteria | Actinomycetota | unclassified_f__Geodermatophilaceae | 22492 | 22880 | 24004 |
| Bacteria | Actinomycetota | Nocardia | 18933 | 19368 | 21276 |
| Bacteria | Actinomycetota | Marmoricola | 17593 | 17743 | 18988 |
| Bacteria | Actinomycetota | Dactylosporangium | 14302 | 14575 | 15603 |
| Bacteria | Actinomycetota | unclassified_o__Frankiales | 14029 | 14711 | 17301 |
| Bacteria | Actinomycetota | Cellulomonas | 13954 | 14190 | 15417 |
| Bacteria | Actinomycetota | Patulibacter | 12847 | 12054 | 13635 |
| Bacteria | Actinomycetota | Actinomycetospora | 12794 | 12081 | 12527 |
| Bacteria | Actinomycetota | Kibdelosporangium | 10998 | 11468 | 16629 |
| Bacteria | Actinomycetota | Georgenia | 9470 | 9599 | 9972 |
| Bacteria | Actinomycetota | unclassified_f__Pseudonocardiaceae | 9353 | 9417 | 10318 |
| Bacteria | Actinomycetota | Aeromicrobium | 8152 | 7998 | 8078 |
| Bacteria | Actinomycetota | unclassified_f__Ilumatobacteraceae | 4688 | 4451 | 4708 |
| Bacteria | Actinomycetota | unclassified_f__Kineosporiaceae | 2317 | 2379 | 2489 |
| Bacteria | Actinomycetota | Promicromonospora | 2199 | 2181 | 2546 |
| Bacteria | Actinomycetota | unclassified_f__Intrasporangiaceae | 655 | 655 | 748 |
| Bacteria | Actinomycetota | Brachybacterium | 582 | 594 | 659 |
| Bacteria | Actinomycetota | unclassified_o__Gaiellales | 394 | 381 | 432 |
| Bacteria | Actinomycetota | Aeromicrobium | 3 | 4 | 4 |
| Bacteria | Actinomycetota | unclassified_f__Thermomonosporaceae | 3 | 1 | 5 |
| Bacteria | Pseudomonadota | Bradyrhizobium | 221392 | 212679 | 238836 |
| Bacteria | Pseudomonadota | Mesorhizobium | 92683 | 90326 | 98698 |
| Bacteria | Pseudomonadota | Roseomonas | 50670 | 42599 | 34132 |
| Bacteria | Pseudomonadota | Belnapia | 38484 | 31669 | 25024 |
| Bacteria | Pseudomonadota | Reyranella | 34504 | 33587 | 36603 |
| Bacteria | Pseudomonadota | unclassified_f__Xanthobacteraceae | 20970 | 18812 | 19161 |
| Bacteria | Pseudomonadota | Bosea | 19334 | 17935 | 15115 |
| Bacteria | Pseudomonadota | Luteimonas | 18343 | 30319 | 26660 |
| Bacteria | Pseudomonadota | Skermanella | 16639 | 14582 | 12309 |
| Bacteria | Pseudomonadota | Geminicoccus | 16150 | 14853 | 13854 |
| Bacteria | Pseudomonadota | Steroidobacter | 11708 | 11708 | 13437 |
| Bacteria | Pseudomonadota | Pseudomonas | 9556 | 9452 | 9676 |
| Bacteria | Pseudomonadota | Pseudorhodoplanes | 8544 | 8009 | 7900 |
| Bacteria | Pseudomonadota | Rhodoplanes | 5744 | 5128 | 4974 |
| Bacteria | Pseudomonadota | unclassified_f__Beijerinckiaceae | 5691 | 5328 | 4836 |
| Bacteria | Pseudomonadota | Archangium | 5098 | 5120 | 5127 |
| Bacteria | Pseudomonadota | Lysobacter | 4220 | 4979 | 6273 |
| Bacteria | Pseudomonadota | Phenylobacterium | 3655 | 3594 | 3492 |
| Bacteria | Pseudomonadota | unclassified_f__Myxococcaceae | 3645 | 3698 | 3697 |
| Bacteria | Pseudomonadota | unclassified_f__Sphingomonadaceae | 3490 | 4166 | 3787 |
| Bacteria | Pseudomonadota | Ramlibacter | 3373 | 3422 | 3497 |
| Bacteria | Pseudomonadota | Devosia | 3183 | 3015 | 3137 |
| Bacteria | Pseudomonadota | Novosphingobium | 2794 | 2889 | 2689 |
| Bacteria | Pseudomonadota | unclassified_f__Rhodobacteraceae | 2775 | 2653 | 2412 |
| Bacteria | Pseudomonadota | Bauldia | 2719 | 2606 | 2793 |
| Bacteria | Pseudomonadota | Rubellimicrobium | 2717 | 2654 | 2114 |
| Bacteria | Pseudomonadota | Massilia | 2208 | 2323 | 2570 |
| Bacteria | Pseudomonadota | Pseudaminobacter | 2173 | 2078 | 2115 |
| Bacteria | Pseudomonadota | Labrys | 1969 | 1818 | 1576 |
| Bacteria | Pseudomonadota | Altererythrobacter | 1627 | 1770 | 2062 |
| Bacteria | Pseudomonadota | unclassified_f__Comamonadaceae | 1526 | 1518 | 1523 |
| Bacteria | Pseudomonadota | Rhodomicrobium | 1327 | 1323 | 1276 |
| Bacteria | Pseudomonadota | Nannocystis | 694 | 732 | 716 |
| Bacteria | Pseudomonadota | Lautropia | 435 | 437 | 410 |
| Bacteria | Pseudomonadota | Haliangium | 341 | 548 | 397 |
| Bacteria | Pseudomonadota | Arenimonas | 295 | 317 | 349 |
| Bacteria | Pseudomonadota | Luteibacter | 277 | 334 | 342 |
| Bacteria | Pseudomonadota | Qipengyuania | 271 | 260 | 251 |
| Bacteria | Pseudomonadota | Vulgatibacter | 197 | 182 | 221 |
| Bacteria | Pseudomonadota | Bradyrhizobium | 77 | 62 | 53 |
| Bacteria | Pseudomonadota | Hirschia | 19 | 13 | 10 |
| Bacteria | Pseudomonadota | Alcaligenes | 18 | 21 | 24 |
| Bacteria | Bacteroidota | Flavisolibacter | 1714.5 | 1553.5 | 1460.75 |
| Bacteria | Bacteroidota | Flavobacterium | 908.75 | 890.5 | 835 |
| Bacteria | Bacteroidota | unclassified_f__Rhodothermaceae | 605.75 | 622.5 | 672 |
| Bacteria | Bacteroidota | unclassified_f__Microscillaceae | 23.75 | 42 | 33.75 |
| Bacteria | Gemmatimonadota | unclassified_f__Gemmatimonadaceae | 71362 | 73534 | 80060 |
| Bacteria | Gemmatimonadota | Gemmatimonas | 4660 | 4713 | 4850 |
| Bacteria | Planctomycetota | Singulisphaera | 8759 | 8309 | 7489 |
| Bacteria | Planctomycetota | unclassified_f__Isosphaeraceae | 3541 | 3479 | 3226 |
| Bacteria | Acidobacteriota | Candidatus_Solibacter | 1371 | 1344 | 1339 |
| Bacteria | Acidobacteriota | Bryobacter | 97 | 103 | 107 |
| Bacteria | unclassified_d__Bacteria | unclassified_d__Bacteria | 101299 | 101493 | 101657 |
| Bacteria | Chloroflexota | unclassified_o__Thermomicrobiales | 35556 | 34464 | 33610 |
| Bacteria | Nitrospirota | Nitrospira | 24591 | 25758 | 25457 |
| Bacteria | Deinococcus-Thermus | Truepera | 1112 | 1085 | 1041 |
| Bacteria | Verrucomicrobiota | Candidatus_Udaeobacter | 758 | 782 | 764 |
| Bacteria | Armatimonadota | unclassified_f__Fimbriimonadaceae | 346 | 325 | 306 |
| Fungi | Ascomycota | Alternaria | 31 | 24 | 16 |
| Fungi | Ascomycota | Cladophialophora | 19 | 18 | 16 |
| Fungi | Ascomycota | Penicillium | 14 | 12 | 13 |
| Fungi | Ascomycota | Trichoderma | 6 | 6 | 2 |
| Fungi | Ascomycota | Paraphaeosphaeria | 0 | 4 | 1 |
| Fungi | Ascomycota | Metarhizium | 4 | 3 | 6 |
| Fungi | Ascomycota | unclassified_o__Chaetothyriales | 0 | 2 | 0 |
| Fungi | Ascomycota | Chaetomium | 2 | 1 | 1 |
| Fungi | Ascomycota | Clonostachys | 0 | 0 | 1 |
| Fungi | Mucoromycota | Mortierella | 25 | 21 | 22 |

Supplementary Table 3. Distribution of glycosyl hydrolase family in the Microorganisms screened out by LDA analysis

| ID | CAZy Family | Organism source | |
| --- | --- | --- | --- |
|  |  | Phylum | Genus |
| 1 | GH1 | Actinomycetota | Actinophytocola |
| 2 | GH1 | Actinomycetota | Actinoplanes |
| 3 | GH1 | Actinomycetota | Aeromicrobium |
| 4 | GH1 | Actinomycetota | Asanoa |
| 5 | GH1 | Actinomycetota | Cellulomonas |
| 6 | GH1 | Actinomycetota | Dactylosporangium |
| 7 | GH1 | Actinomycetota | Georgenia |
| 8 | GH1 | Actinomycetota | Kibdelosporangium |
| 9 | GH1 | Actinomycetota | Microlunatus |
| 10 | GH1 | Actinomycetota | unclassified_c__Acidimicrobiia |
| 11 | GH1 | Actinomycetota | unclassified_c__Thermoleophilia |
| 12 | GH1 | Actinomycetota | unclassified_f__Micromonosporaceae |
| 13 | GH1 | Bacteroidota | Flavisolibacter |
| 14 | GH1 | Chloroflexota | unclassified_o__Thermomicrobiales |
| 15 | GH1 | Gemmatimonadota | unclassified_f__Gemmatimonadaceae |
| 16 | GH1 | Nitrospirota | Nitrospira |
| 17 | GH1 | Planctomycetota | unclassified_f__Isosphaeraceae |
| 18 | GH1 | Pseudomonadota | Archangium |
| 19 | GH1 | Pseudomonadota | Belnapia |
| 20 | GH1 | Pseudomonadota | Bradyrhizobium |
| 21 | GH1 | Pseudomonadota | Bradyrhizobium |
| 22 | GH1 | Pseudomonadota | Devosia |
| 23 | GH1 | Pseudomonadota | Luteimonas |
| 24 | GH1 | Pseudomonadota | Lysobacter |
| 25 | GH1 | Pseudomonadota | Massilia |
| 26 | GH1 | Pseudomonadota | Mesorhizobium |
| 27 | GH1 | Pseudomonadota | Phenylobacterium |
| 28 | GH1 | Pseudomonadota | Pseudaminobacter |
| 29 | GH1 | Pseudomonadota | Pseudomonas |
| 30 | GH1 | Pseudomonadota | Ramlibacter |
| 31 | GH1 | Pseudomonadota | Reyranella |
| 32 | GH1 | Pseudomonadota | Roseomonas |
| 33 | GH1 | Pseudomonadota | Rubellimicrobium |
| 34 | GH1 | Pseudomonadota | Skermanella |
| 35 | GH1 | Pseudomonadota | Steroidobacter |
| 36 | GH1 | Pseudomonadota | unclassified_f__Myxococcaceae |
| 37 | GH1 | Pseudomonadota | unclassified_f__Rhodobacteraceae |
| 38 | GH1 | Pseudomonadota | unclassified_f__Sphingomonadaceae |
| 39 | GH1 | unclassified_d__Bacteria | unclassified_d__Bacteria |
| 40 | GH5_7 | Bacteroidota | unclassified_f__Rhodothermaceae |
| 41 | GH5_7 | Pseudomonadota | unclassified_f__Sphingomonadaceae |
| 42 | GH6 | Actinomycetota | Actinophytocola |
| 43 | GH6 | Actinomycetota | Actinoplanes |
| 44 | GH6 | Actinomycetota | Aeromicrobium |
| 45 | GH6 | Actinomycetota | Asanoa |
| 46 | GH6 | Actinomycetota | Marmoricola |
| 47 | GH6 | Actinomycetota | Microlunatus |
| 48 | GH6 | Actinomycetota | Patulibacter |
| 49 | GH6 | Actinomycetota | unclassified_c__Thermoleophilia |
| 50 | GH6 | Actinomycetota | unclassified_f__Micromonosporaceae |
| 51 | GH6 | Chloroflexota | unclassified_o__Thermomicrobiales |
| 52 | GH6 | Gemmatimonadota | unclassified_f__Gemmatimonadaceae |
| 53 | GH6 | Pseudomonadota | Steroidobacter |
| 54 | GH6 | unclassified_d__Bacteria | unclassified_d__Bacteria |
| 55 | GH13_1 | Actinomycetota | Actinoplanes |
| 56 | GH15 | Actinomycetota | Actinomycetospora |
| 57 | GH15 | Actinomycetota | Actinophytocola |
| 58 | GH15 | Actinomycetota | Actinoplanes |
| 59 | GH15 | Actinomycetota | Aeromicrobium |
| 60 | GH15 | Actinomycetota | Aeromicrobium |
| 61 | GH15 | Actinomycetota | Asanoa |
| 62 | GH15 | Actinomycetota | Cellulomonas |
| 63 | GH15 | Actinomycetota | Dactylosporangium |
| 64 | GH15 | Actinomycetota | Georgenia |
| 65 | GH15 | Actinomycetota | Kibdelosporangium |
| 66 | GH15 | Actinomycetota | Microlunatus |
| 67 | GH15 | Actinomycetota | Nocardia |
| 68 | GH15 | Actinomycetota | Patulibacter |
| 69 | GH15 | Actinomycetota | Promicromonospora |
| 70 | GH15 | Actinomycetota | unclassified_c__Acidimicrobiia |
| 71 | GH15 | Actinomycetota | unclassified_c__Thermoleophilia |
| 72 | GH15 | Actinomycetota | unclassified_f__Micromonosporaceae |
| 73 | GH15 | Chloroflexota | unclassified_o__Thermomicrobiales |
| 74 | GH15 | Gemmatimonadota | unclassified_f__Gemmatimonadaceae |
| 75 | GH15 | Nitrospirota | Nitrospira |
| 76 | GH15 | Planctomycetota | Singulisphaera |
| 77 | GH15 | Planctomycetota | unclassified_f__Isosphaeraceae |
| 78 | GH15 | Pseudomonadota | Archangium |
| 79 | GH15 | Pseudomonadota | Belnapia |
| 80 | GH15 | Pseudomonadota | Bradyrhizobium |
| 81 | GH15 | Pseudomonadota | Bradyrhizobium |
| 82 | GH15 | Pseudomonadota | Geminicoccus |
| 83 | GH15 | Pseudomonadota | Lautropia |
| 84 | GH15 | Pseudomonadota | Luteimonas |
| 85 | GH15 | Pseudomonadota | Mesorhizobium |
| 86 | GH15 | Pseudomonadota | Phenylobacterium |
| 87 | GH15 | Pseudomonadota | Pseudomonas |
| 88 | GH15 | Pseudomonadota | Pseudorhodoplanes |
| 89 | GH15 | Pseudomonadota | Ramlibacter |
| 90 | GH15 | Pseudomonadota | Reyranella |
| 91 | GH15 | Pseudomonadota | Roseomonas |
| 92 | GH15 | Pseudomonadota | Skermanella |
| 93 | GH15 | Pseudomonadota | Steroidobacter |
| 94 | GH15 | Pseudomonadota | unclassified_f__Comamonadaceae |
| 95 | GH15 | Pseudomonadota | unclassified_f__Myxococcaceae |
| 96 | GH15 | unclassified_d__Bacteria | unclassified_d__Bacteria |
| 97 | GH23 | Actinomycetota | Actinomycetospora |
| 98 | GH23 | Actinomycetota | Actinophytocola |
| 99 | GH23 | Actinomycetota | Actinoplanes |
| 100 | GH23 | Actinomycetota | Aeromicrobium |
| 101 | GH23 | Actinomycetota | Asanoa |
| 102 | GH23 | Actinomycetota | Dactylosporangium |
| 103 | GH23 | Actinomycetota | Kibdelosporangium |
| 104 | GH23 | Actinomycetota | Marmoricola |
| 105 | GH23 | Actinomycetota | Microlunatus |
| 106 | GH23 | Actinomycetota | Nocardia |
| 107 | GH23 | Actinomycetota | Patulibacter |
| 108 | GH23 | Actinomycetota | unclassified_c__Acidimicrobiia |
| 109 | GH23 | Actinomycetota | unclassified_c__Acidimicrobiia |
| 110 | GH23 | Actinomycetota | unclassified_c__Thermoleophilia |
| 111 | GH23 | Actinomycetota | unclassified_f__Micromonosporaceae |
| 112 | GH23 | Actinomycetota | unclassified_o__Frankiales |
| 113 | GH23 | Armatimonadota | unclassified_f__Fimbriimonadaceae |
| 114 | GH23 | Chloroflexota | unclassified_o__Thermomicrobiales |
| 115 | GH23 | Gemmatimonadota | Gemmatimonas |
| 116 | GH23 | Gemmatimonadota | unclassified_f__Gemmatimonadaceae |
| 117 | GH23 | Nitrospirota | Nitrospira |
| 118 | GH23 | Pseudomonadota | Altererythrobacter |
| 119 | GH23 | Pseudomonadota | Archangium |
| 120 | GH23 | Pseudomonadota | Belnapia |
| 121 | GH23 | Pseudomonadota | Bradyrhizobium |
| 122 | GH23 | Pseudomonadota | Bradyrhizobium |
| 123 | GH23 | Pseudomonadota | Geminicoccus |
| 124 | GH23 | Pseudomonadota | Luteimonas |
| 125 | GH23 | Pseudomonadota | Lysobacter |
| 126 | GH23 | Pseudomonadota | Mesorhizobium |
| 127 | GH23 | Pseudomonadota | Pseudomonas |
| 128 | GH23 | Pseudomonadota | Pseudorhodoplanes |
| 129 | GH23 | Pseudomonadota | Reyranella |
| 130 | GH23 | Pseudomonadota | Roseomonas |
| 131 | GH23 | Pseudomonadota | Skermanella |
| 132 | GH23 | Pseudomonadota | Steroidobacter |
| 133 | GH23 | Pseudomonadota | unclassified_f__Sphingomonadaceae |
| 134 | GH23 | Pseudomonadota | unclassified_f__Xanthobacteraceae |
| 135 | GH23 | unclassified_d__Bacteria | unclassified_d__Bacteria |
| 136 | GH46 | Actinomycetota | Actinoplanes |
| 137 | GH46 | Actinomycetota | Georgenia |
| 138 | GH46 | Actinomycetota | Kibdelosporangium |
| 139 | GH46 | Actinomycetota | unclassified_f__Micromonosporaceae |
| 140 | GH94 | Actinomycetota | Actinoplanes |
| 141 | GH94 | Actinomycetota | Aeromicrobium |
| 142 | GH94 | Actinomycetota | Aeromicrobium |
| 143 | GH94 | Actinomycetota | Dactylosporangium |
| 144 | GH94 | Actinomycetota | unclassified_c__Acidimicrobiia |
| 145 | GH94 | Actinomycetota | unclassified_c__Thermoleophilia |
| 146 | GH94 | Actinomycetota | unclassified_f__Ilumatobacteraceae |
| 147 | GH94 | Actinomycetota | unclassified_f__Micromonosporaceae |
| 148 | GH94 | Gemmatimonadota | Gemmatimonas |
| 149 | GH94 | Gemmatimonadota | unclassified_f__Gemmatimonadaceae |
| 150 | GH94 | Nitrospirota | Nitrospira |
| 151 | GH94 | Planctomycetota | Singulisphaera |
| 152 | GH94 | Planctomycetota | unclassified_f__Isosphaeraceae |
| 153 | GH94 | Pseudomonadota | Luteibacter |
| 154 | GH94 | Pseudomonadota | Luteimonas |
| 155 | GH94 | Pseudomonadota | Lysobacter |
| 156 | GH94 | Pseudomonadota | Massilia |
| 157 | GH94 | Pseudomonadota | Mesorhizobium |
| 158 | GH94 | Pseudomonadota | Ramlibacter |
| 159 | GH94 | Pseudomonadota | Reyranella |
| 160 | GH94 | Pseudomonadota | Rhodomicrobium |
| 161 | GH94 | Pseudomonadota | Skermanella |
| 162 | GH94 | Pseudomonadota | Steroidobacter |
| 163 | GH94 | Pseudomonadota | unclassified_f__Myxococcaceae |
| 164 | GH94 | unclassified_d__Bacteria | unclassified_d__Bacteria |
| 165 | GH114 | Actinomycetota | Actinophytocola |
| 166 | GH114 | Actinomycetota | Actinoplanes |
| 167 | GH114 | Actinomycetota | Nocardia |
| 168 | GH114 | Actinomycetota | unclassified_c__Thermoleophilia |
| 169 | GH114 | Actinomycetota | unclassified_f__Micromonosporaceae |
| 170 | GH114 | Pseudomonadota | Bauldia |
| 171 | GH114 | Pseudomonadota | Devosia |
| 172 | GH114 | Pseudomonadota | Lysobacter |
| 173 | GH114 | Pseudomonadota | Mesorhizobium |
| 174 | GH114 | Pseudomonadota | Roseomonas |
| 175 | GH116 | Actinomycetota | Microlunatus |
| 176 | GH116 | Chloroflexota | unclassified_o__Thermomicrobiales |
| 177 | GH116 | Gemmatimonadota | unclassified_f__Gemmatimonadaceae |
| 178 | GH116 | Planctomycetota | Singulisphaera |
| 179 | GH116 | Pseudomonadota | Bradyrhizobium |
| 180 | GH116 | Pseudomonadota | Bradyrhizobium |
| 181 | GH116 | Pseudomonadota | Reyranella |
| 182 | GH116 | unclassified_d__Bacteria | unclassified_d__Bacteria |
| 183 | GH138 | Actinomycetota | Microlunatus |
| 184 | GH138 | Bacteroidota | Flavisolibacter |
| 185 | GT10 | unclassified_d__Bacteria | unclassified_d__Bacteria |
| 186 | GT46 | Actinomycetota | Actinophytocola |
| 187 | GT46 | Actinomycetota | Kibdelosporangium |
| 188 | GT46 | Nitrospirota | Nitrospira |
| 189 | GT46 | Pseudomonadota | Bradyrhizobium |
| 190 | GT46 | Pseudomonadota | Bradyrhizobium |
| 191 | GT46 | Pseudomonadota | Pseudomonas |
| 192 | GT46 | Pseudomonadota | Roseomonas |
| 193 | CBM22 | Actinomycetota | Actinophytocola |
| 194 | CBM22 | Actinomycetota | Actinoplanes |
| 195 | CBM22 | Actinomycetota | Cellulomonas |
| 196 | CBM22 | Pseudomonadota | Pseudomonas |
